# Global Burden of Pediatric Rheumatic Heart Disease, 1990–2021: Analysis of the GBD 2021 Study

**DOI:** 10.3390/children12070843

**Published:** 2025-06-26

**Authors:** Ze Tang, Ziwei Wang, Xinbao Wang

**Affiliations:** Department of Pediatrics, Beijing Friendship Hospital, Capital Medical University, Beijing 100050, China

**Keywords:** rheumatic heart disease, Global Burden of Disease, children, disability-adjusted life years (DALYs), health inequality

## Abstract

**Background**: Rheumatic heart disease (RHD) remains a major contributor to childhood cardiovascular morbidity and mortality globally, particularly in low-resource settings. This study offers a thorough evaluation of the global, regional, and national burden of RHD among children aged 0–14 years, from 1990 to 2021, utilizing data from the 2021 Global Burden of Disease (GBD) study. **Methods**: We analyzed age-standardized incidence, prevalence, mortality, and disability-adjusted life years (DALYs) for RHD in 204 countries and territories. Novel methodological approaches included APC analysis to decompose temporal trends into age, period, and cohort effects, and inequality analysis to assess socioeconomic disparities. We calculated age-standardized rates and average annual percentage changes (AAPC) by sex, region, and socio-demographic index (SDI) level. **Results**: From 1990 to 2021, the global age-standardized death rate due to RHD in children declined by approximately 74%, from 1.24 to 0.32 per 100,000 (AAPC: −4.27%). Similarly, DALY rates dropped from 117.22 to 41.56 per 100,000 (AAPC: −3.30%). Despite this progress, the global age-standardized incidence rate increased modestly from 55.84 to 66.76 per 100,000 (AAPC: 0.58%), and prevalence rates also rose (AAPC: 0.53%). Females consistently experienced higher burden across all metrics. Inequality analysis demonstrated a concerning divergence: while mortality and DALY inequalities narrowed substantially (mortality slope index of inequality (SII) improved from −1.35 to −0.31), incidence and prevalence inequalities widened (incidence SII worsened from −112.60 to −131.90), indicating growing disparities in disease occurrence despite improved survival. **Conclusions**: While global mortality and DALYs from childhood rheumatic heart disease have declined substantially over the past three decades, a troubling paradox has emerged: rising incidence rates alongside widening socioeconomic inequalities in disease occurrence. This represents a critical public health challenge demanding targeted intervention strategies. The divergent trends in health outcomes, namely, improved survival rates but increased disease burden, reveal that while access to treatment has advanced, upstream prevention efforts remain critically inadequate among socioeconomically disadvantaged populations.

## 1. Introduction

Rheumatic heart disease (RHD) remains a major yet avoidable contributor to global cardiovascular illness and death rates. It is a chronic consequence of acute rheumatic fever, an autoimmune reaction to inadequately treated group A streptococcal (GAS) pharyngitis, resulting in permanent damage to the heart valves [1]. Although significant progress has been made in high-income countries through the improvement of living conditions, widespread antibiotic use, and robust healthcare systems, RHD remains a significant healthcare burden across numerous developing nations with limited resources [2,3]. Recent estimates suggest over 40 million people live with RHD globally, a disease which causes more than 300,000 deaths annually [4,5]. However, many cases remain undiagnosed due to lack of systematic screening, especially among children in high-risk settings [6].

Children and adolescents are particularly vulnerable. Initial GAS infections frequently occur during early childhood, and recurrent episodes increase the risk of rheumatic fever and irreversible cardiac damage. Children aged 5–14 years are within a critical window of risk, as disease symptoms often emerge during this period, leading to lifelong disability and increased healthcare costs [7,8]. RHD also reflects deeper structural inequities, including overcrowding, poor hygiene, and inadequate access to primary care [9]. Despite being entirely preventable, RHD is among the most neglected cardiovascular diseases in global child health policy and remains excluded from many national non-communicable disease agendas [8,9]. Improved diagnostic technologies such as echocardiography have enhanced disease detection in the last decade, but implementation at scale remains limited [3].

The Global Burden of Disease (GBD) study has substantially advanced the understanding of RHD epidemiology over the past decade, yet gaps persist. Earlier assessments lacked sufficient age-specific granularity, relied on sparse data, and exhibited methodological inconsistencies across time and geography [5,10]. Comprehensive analyses specific to children aged 0–14 years have been missing.

This research seeks to bridge existing knowledge gaps through a comprehensive evaluation of RHD impacts across different age groups (0–14 years), worldwide, regionally, and nationally, between 1990 and 2021. Utilizing methodological approaches and datasets from the GBD 2021 study, we analyze temporal patterns in disease occurrence, affected populations, fatal outcomes, and disability-adjusted life years (DALYs). Additionally, we investigate correlations with socioeconomic development metrics (SDI) and healthcare disparity measures to pinpoint inequities and guide focused public health strategies.

## 2. Methods

### 2.1. Overview

The research utilizes an extensive evaluation methodology embedded in the GBD structure to examine worldwide RHD impacts exclusively in pediatric populations (0–14 years) during 1990–2021. Key aims involve measuring standardized age-related metrics for disease occurrence, affected cases, fatality rates, and DALYs, evaluating geographic and temporal distribution patterns, analyzing socio-demographic influences, and exploring inequalities in disease burden (Figure S1 in the Supplemental Material).

### 2.2. Data Sources

Key statistics were sourced directly from the 2021 GBD research repositories (https://vizhub.healthdata.org/gbd-results/, accessed on 9 April 2025) [11], which consolidate epidemiological estimates from global surveillance systems, national registries, hospital databases, and the published literature [12,13,14]. Supplementary data from the World Health Organization (WHO) was used to enhance data completeness and accuracy [15].

### 2.3. Disease Burden Metrics

Key disease burden metrics analyzed include incidence (newly diagnosed cases per year), prevalence (total existing cases per year), deaths attributed directly to RHD, and DALYs. Incidence and prevalence provide essential epidemiological insights into disease frequency, while deaths and DALYs quantify the health and societal impacts comprehensively, facilitating targeted policy interventions and healthcare planning [4].

### 2.4. Distribution and Trends Analysis

Geographical distribution, temporal trends, and regional variations in pediatric RHD burden were evaluated using standardized age-specific rates per 100,000 population. Descriptive analyses visualized global patterns using maps, while trends over time were assessed using segment regression analyses to detect statistically significant temporal inflection points [16].

### 2.5. Socio-Demographic Index (SDI)

The SDI served as an integrated metric used for classifying nations according to three key indicators: economic output per person, average education level, and birth rate. This framework divided countries into five distinct SDI tiers (from the lowest to the highest level of development), enabling analysis of how societal progress correlates with health outcomes. The stratification ranged from low-development to high-development groups, providing insights into socioeconomic influences on public health challenges.

### 2.6. AAPC Analysis

Our analysis employed piecewise linear regression modeling to evaluate the annual average percentage change (AAPC) for age-adjusted incidence rates across the 1990–2021 timeframe. Log-linear models were fitted with year as the independent variable. Joinpoints were identified using the segmented package in R. The percentage variation per annum was computed for every market segment, with the AAPC being determined through a time-weighted aggregation of yearly percentage fluctuations. Statistical significance was determined using 95% confidence intervals [17,18].

### 2.7. Age–Period–Cohort (APC) Analysis

Our investigation employed age–period–cohort (APC) modeling to differentiate the distinct influences of chronological age, time period, and generational cohort on disease prevalence and fatality rates. The National Cancer Institute-endorsed NIH APC Web Tool (https://analysistools.cancer.gov/apc/, accessed on 9 April 2025) facilitated our computations, specifically, by generating net drift measurements—representing the yearly percentage variation in disease metrics after accounting for cohort-specific impacts, local drift (age-specific EAPC), and relative risks (RRs) for period and cohort. Both longitudinal and cross-sectional age curves were derived. The significance of local drift was tested to evaluate age–cohort interactions [19].

### 2.8. Health Inequality Analysis

Disparities in health outcomes were measured through two distinct metrics: the Gradient Disparity Indicator and the Inequality Concentration Metric. The analysis employed these complementary indices to assess socioeconomic gradients in population health status, with particular attention to relative distribution patterns across different demographic strata. These quantitative measures facilitated the examination of systematic variations in health outcomes along the socioeconomic spectrum (concentration Index of Inequality, CII). The slope index of inequality (SII) quantified absolute inequalities, capturing differences in RHD burden across the socioeconomic spectrum, while the CII measured relative inequalities, assessing how disease burden concentration varied across socioeconomic groups. Both indices were computed using regression-based approaches adjusted for demographic confounders [20,21].

### 2.9. Statistical Software and Significance

The statistical computations were performed with R programming environment release 4.4.2 (developed by R Core Team, headquartered in Vienna, Austria). A threshold of *p* < 0.05 in two-sided hypothesis testing was established as the criterion for determining statistical significance, with 95% uncertainty intervals (UI) or 95% confidence interval (CI) presented alongside all estimates to reflect measurement uncertainty.

### 2.10. Ethical Approval and Reporting Standards

No ethical clearance was necessary, since this investigation employed pre-existing, compiled datasets from GBD research containing no personally identifiable health records. 

## 3. Results

### 3.1. Global Trend

From 1990 to 2021, the global age-standardized death rate (ASDR) due to RHD among children aged 0–14 years declined significantly, reflecting notable improvements in child health outcomes. Globally, the ASDR dropped from 1.24 (95% UI: 0.95–1.64) per 100,000 in 1990 to 0.32 (95% UI: 0.25–0.39) in 2021, with an average annual percentage change (AAPC) of −4.27% (95% CI: −4.45 to −4.09) (Table 1). Globally, the age-standardized rate (ASR) of DALYs fell from 117.22 (95% UI: 90.07–154.09) per 100,000 population in 1990 to 41.56 (95% UI: 31.65–54.84) in 2021, with a corresponding AAPC of −3.30% (95% CI: −3.41 to −3.18), demonstrating a statistically significant downward trend (Table S1 in Supplemental Material).

From 1990 through 2021, researchers observed a slight yet meaningful rise in the worldwide age-standardized incidence rate (ASIR) of RHD for pediatric populations under 15 years old, rising from 55.84 (95% UI: 30.74–89.59) to 66.76 (95% UI: 36.40–107.60) per 100,000 population, with an AAPC of 0.58% (95% CI: 0.51–0.65) (Table 2). Globally, the number of RHD cases increased from 4.37 million (95% UI: 3.00–6.07 million) in 1990 to 6.23 million (95% UI: 4.24–8.73 million) in 2021, with a corresponding rise in age-standardized prevalence rate (ASPR) from 253.82 (95% UI: 167.38–366.88) to 298.55 (95% UI: 194.94–435.24) per 100,000 population (AAPC: 0.53%; 95% CI: 0.45–0.62) (Table S2 in Supplemental Material).

### 3.2. SDI Trend

#### 3.2.1. Mortality

**Figure 1:**
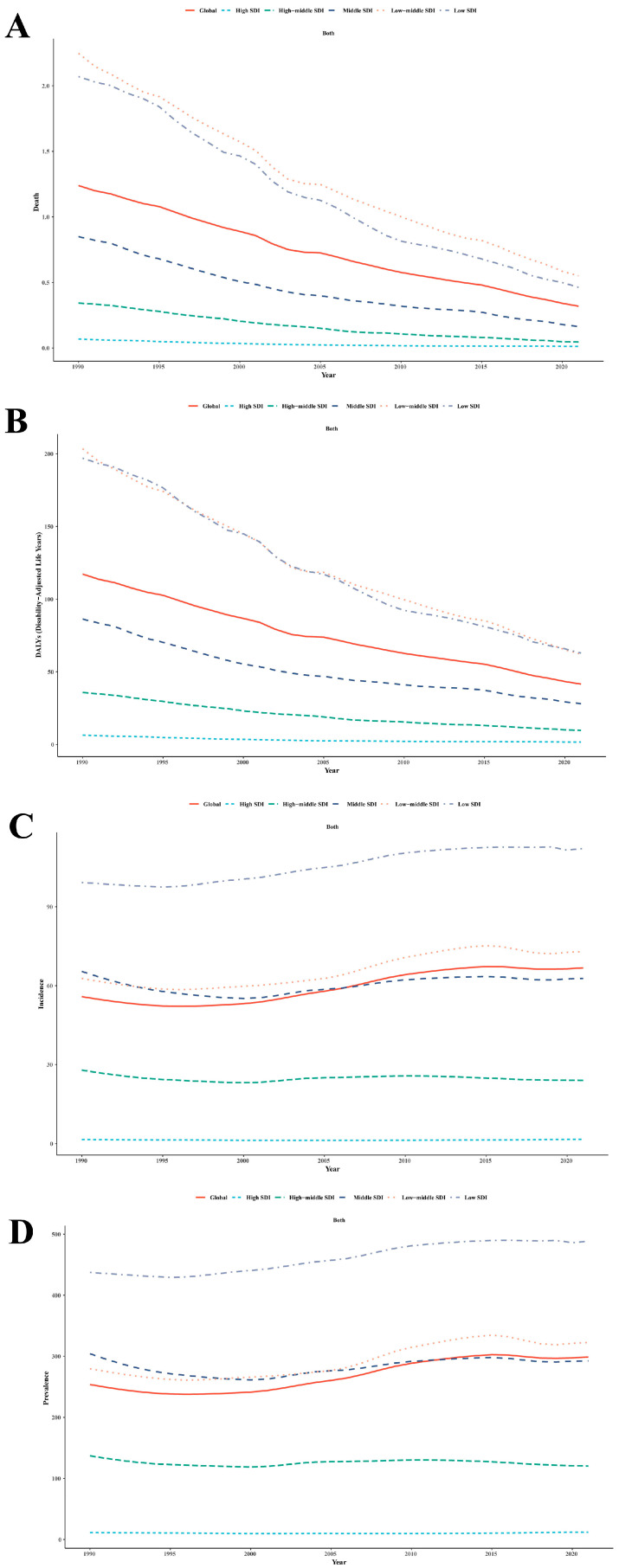
Trends in age-standardized (**A**) death, (**B**) disability-adjusted life years (DALYs), (**C**) incidence, and (**D**) prevalence rates associated with rheumatic heart disease among children aged 0–14 years, by SDI level, 1990–2021.

From 1990 to 2021, global pediatric RHD mortality (ages 0–14) declined significantly, though SDI disparities persisted (Figure 1A). In 1990, low-SDI regions had the highest ASDR, at 2.07 (95% UI: 1.39–3.00), followed by low-middle SDI at 2.25 (95% UI: 1.67–3.06). By 2021, both showed substantial reductions (low-SDI: 0.46; 95% UI: 0.32–0.64; low-middle SDI: 0.55; 95% UI: 0.43–0.69), yet remained disproportionately high compared to high-SDI regions, which decreased from 0.07 (95% UI: 0.06–0.08) to 0.01 (95% UI: 0.01–0.01). High-middle-SDI countries also improved markedly, from 0.34 (95% UI: 0.29–0.42) to 0.045 (95% UI: 0.037–0.055).

#### 3.2.2. DALYs

Between 1990 and 2021, global pediatric RHD DALYs declined significantly across all SDI regions, though disparities persisted (Figure 1B). By 2021, high-SDI countries achieved the lowest burden, at 1.69 (95% UI: 1.39–2.08), compared to 9.77 (95% UI: 6.88–13.74) in high-middle SDI, 28.09 (95% UI: 20.93–38.52) in middle SDI, 62.24 (95% UI: 48.86–79.33) in low-middle SDI, and 62.84 (95% UI: 44.01–88.03) in low-SDI regions. Low and low-middle-SDI regions showed slower reductions. Low-SDI regions declined from 196.97 (95% UI: 135.51–279.97) in 1990 to 62.84 in 2021.

#### 3.2.3. Incidence

From 1990 to 2021, global pediatric RHD incidence rates (ages 0–14) showed a statistically significant slight increase (Figure 1C). Low-SDI countries experienced a rising ASIR, from 99.21 (95% UI: 53.37–159.11) to 112.14 (95% UI: 60.73–180.56), while low-middle-SDI regions increased from 62.73 (95% UI: 33.84–101.46) to 72.97 (95% UI: 39.22–117.42). High-SDI regions remained consistently low, rising minimally from 1.61 (95% UI: 1.07–2.23) to 1.68 (95% UI: 1.10–2.34). High-middle-SDI regions showed steady decline from 27.88 (95% UI: 16.05–43.56) to 24.00 (95% UI: 13.61–37.99), suggesting improved prevention and early detection. Middle-SDI countries fluctuated but ended at 62.75 (95% UI: 34.64–100.51) in 2021.

#### 3.2.4. Prevalence

Between 1990 and 2021, global pediatric RHD prevalence increased moderately, with significant SDI variation (Figure 1D). Low-SDI countries maintained the highest burden, rising from 437.23 (95% UI: 284.35–635.09) to 488.29 (95% UI: 317.07–718.84). Low-middle-SDI regions increased from 279.54 (95% UI: 181.43–406.50) to 322.33 (95% UI: 207.96–470.50), reflecting persistent high prevalence. High-SDI countries maintained the lowest prevalence, rising slightly from 11.25 (95% UI: 8.67–14.09) to 11.84 (95% UI: 9.17–14.82). High-middle-SDI regions showed decline from 136.97 (95% UI: 93.98–193.39) to 120.21 (95% UI: 81.96–171.06). Middle-SDI regions experienced a modest change from 304.17 (95% UI: 200.75–438.41) to 292.36 (95% UI: 191.89–424.33).

### 3.3. Sex and Region

**Figure 2:**
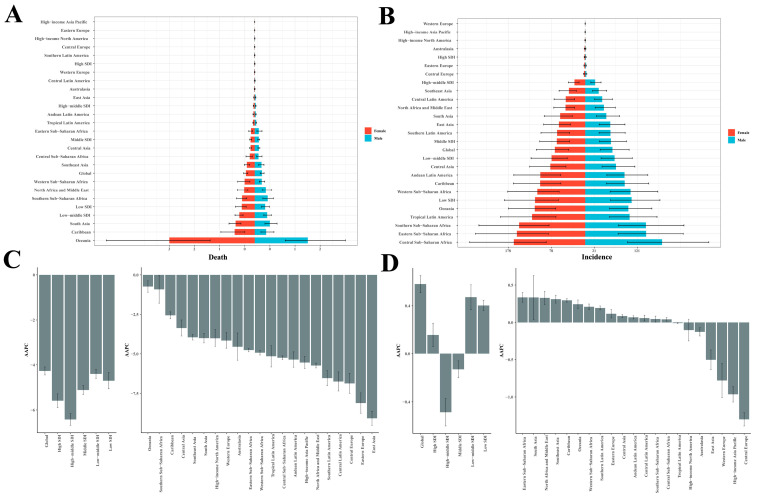
Age-standardized (**A**) death rates and (**B**) incidence rates of rheumatic heart disease among children aged 0–14 years, by sex and region, 2021. The annual average percentage change (AAPC) in mortality (**C**) and the incidence (**D**) rates of rheumatic heart disease are observed in pediatric populations (0–14 years) across different geographical zones, from 1990 through 2021.

In 2021, pediatric RHD cases (ages 0–14) showed significant gender and geographical variations. Globally, ASDR values were higher in girls (0.35 per 100,000; 95% UI: 0.27–0.44) than boys (0.29; 95% UI: 0.22–0.37) (Figure 2A). Oceania exhibited the highest ASDR (female: 3.40 [1.77–5.90]; male: 2.11 [1.21–3.61]), while affluent regions showed minimal rates. Gender mortality gaps were most pronounced in lower-middle-SDI nations. DALY patterns mirrored mortality, with global rates favoring females (44.68; 95% UI: 33.89–59.06 versus male: 38.63; 95% UI: 28.42–51.57) (Figure S2 in Supplemental Material). Oceania remained most affected, while high-SDI zones showed negligible burdens. Incidence rates globally favored females (70.40 per 100,000; 95% UI: 38.19–113.48) over males (63.35; 95% UI: 34.75–102.05), with Sub-Saharan Africa and Oceania showing peak values (Figure 2B). Low-SDI territories had the highest incidence burdens. Prevalence patterns were similar, with females showing higher worldwide rates (312.23; 95% UI: 203.55–454.54 versus male: 285.71; 95% UI: 186.63–416.29). Central Sub-Saharan Africa registered extreme prevalence values (Figure S3 in the Supplemental Material).

#### Crowd Analysis

**Figure 3:**
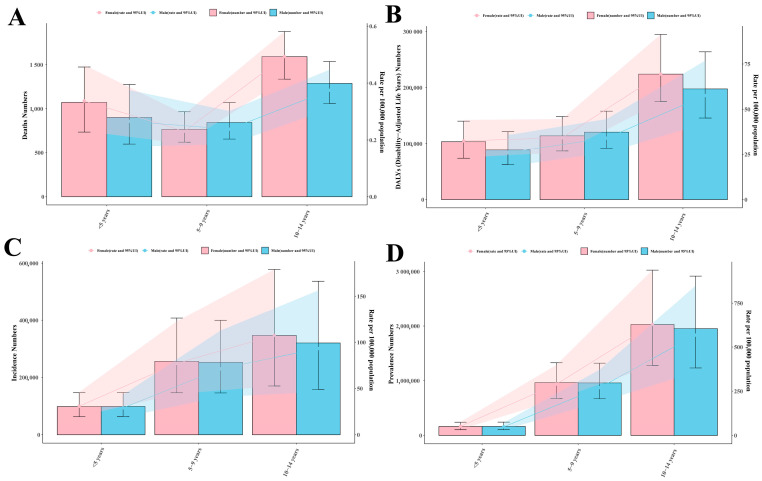
Global distribution of RHD-associated (**A**) mortality, (**B**) DALY burden, (**C**) incidence, and (**D**) prevalence, among children aged 0–14 years, and as stratified by age groups and biological sex (2021).

In 2021, the pediatric RHD burden showed distinct age and gender patterns. Death rates increased with age, from 0.27 (95% UI: 0.18–0.38) per 100,000 in males and 0.34 (95% UI: 0.23–0.46) in females aged <5 years, to 0.49 (95% UI: 0.41–0.58) in females and 0.37 (95% UI: 0.31–0.45) in males aged 10–14 years (Figure 3A). Total deaths peaked in the 10–14 age group: 1591 (95% UI: 1336–1877) females and 1288 (95% UI: 1059–1535) males. DALYs followed similar patterns, rising from 32.52 (95% UI: 23.20–44.06) per 100,000 in females <5 years to 69.43 (95% UI: 54.21–91.34) in those 10–14 years (Figure 3B). Males increased from 26.09 (95% UI: 18.33–35.74) to 57.54 (95% UI: 42.39–76.77). Incidence rates rose with age: females from 30.79 (95% UI: 19.68–46.02) per 100,000 in <5 years to 107.44 (95% UI: 52.64–179.04) in 10–14 years; males from 28.99 (95% UI: 18.65–43.23) to 93.16 (95% UI: 45.94–156.23) (Figure 3C). Prevalence peaked in 10–14 year-old: 628.10 (95% UI: 395.91–937.16) in females, versus 50.80 (95% UI: 32.58–75.52) at <5 years; males showed 567.79 (95% UI: 358.46–848.68) versus 47.81 (95% UI: 30.82–71.11). As to prevalence, over 2 million cases occurred in girls and nearly 2 million in boys for the population aged 10–14 (Figure 3D).

### 3.4. Regional Trends

#### 3.4.1. Mortality

From 1990 to 2021, the global ASDR due to RHD among children aged 0–14 years declined significantly, reflecting notable improvements in child health outcomes. Regional disparities remain evident; in 2021, Oceania exhibited the highest ASDR, at 2.72 (95% UI: 1.69–4.12), despite a modest decline (AAPC: −0.73%; 95% CI: −1.09 to −0.37), while Western Europe maintained the lowest ASDR, at 0.01 (95% UI: 0.01–0.01). The greatest absolute reductions in ASDR were observed in East Asia (from 0.64 to 0.03 per 100,000; AAPC: −9.06%; 95% CI: −9.51 to −8.62) and Eastern Europe (AAPC: −8.10%; 95% CI: −8.74 to −7.46). Despite improvements, Sub-Saharan African regions (e.g., Western and Central Sub-Saharan Africa) continue to report elevated mortality, underscoring the persistent health inequities that demand strengthened prevention and treatment strategies (Table 1, Figure 2C).

**Table 1:** Age-standardized death rates (ASDR) for rheumatic heart disease among children aged 0–14 years, global numbers and as categorized by region and SDI level, 1990–2021.

Location	1990 Cases	1990 ASDR (per 100,000, 95% UI)	2021 Cases	2021 ASDR (per 100,000, 95% UI)	AAPC (%, 95% CI)
Global	21,636.42 (17,028.38, 27,998.59)	1.24 (0.95, 1.64)	6465.27 (5289.96, 7791.63)	0.32 (0.25, 0.39)	−4.27 (−4.45, −4.09)
High SDI	125.77 (106.41, 152.88)	0.07 (0.06, 0.08)	20.19 (18.24, 22.40)	0.01 (0.01, 0.01)	−5.59 (−5.89, −5.29)
High-middle SDI	937.39 (810.46, 1123.91)	0.34 (0.29, 0.42)	106.88 (90.13, 125.67)	0.05 (0.04, 0.06)	−6.42 (−6.68, −6.16)
Middle SDI	4903.49 (4198.41, 5654.46)	0.85 (0.71, 1.00)	963.63 (807.55, 1113.94)	0.16 (0.13, 0.19)	−5.12 (−5.31, −4.92)
Low-middle SDI	10,699.14 (8312.89, 14051.33)	2.25 (1.67, 3.06)	3227.40 (2608.62, 3932.28)	0.55 (0.43, 0.69)	−4.40 (−4.60, −4.21)
Low SDI	4953.63 (3435.90, 7137.75)	2.07 (1.39, 3.00)	2136.88 (1546.24, 2877.39)	0.46 (0.32, 0.64)	−4.70 (−5.06, −4.35)
Andean Latin America	35.69 (28.37, 46.27)	0.24 (0.18, 0.33)	8.25 (6.51, 10.55)	0.04 (0.03, 0.06)	−5.36 (−5.84, −4.88)
Australasia	2.88 (2.58, 3.22)	0.06 (0.05, 0.07)	0.84 (0.72, 0.96)	0.01 (0.01, 0.02)	−4.53 (−5.40, −3.67)
Caribbean	162.75 (121.21, 215.74)	1.42 (0.94, 2.00)	72.21 (48.60, 96.22)	0.62 (0.38, 0.93)	−2.55 (−2.77, −2.33)
Central Asia	119.28 (108.18, 131.32)	0.48 (0.43, 0.55)	45.17 (38.33, 52.95)	0.16 (0.13, 0.20)	−3.35 (−3.86, −2.84)
Central Europe	25.26 (23.78, 26.84)	0.08 (0.08, 0.09)	1.53 (1.30, 1.73)	0.01 (0.01, 0.01)	−6.86 (−7.49, −6.23)
Central Latin America	96.67 (91.56, 102.78)	0.15 (0.14, 0.16)	10.35 (8.59, 12.00)	0.02 (0.01, 0.02)	−6.74 (−7.34, −6.14)
Central Sub-Saharan Africa	245.63 (145.05, 381.16)	0.91 (0.43, 1.48)	100.53 (64.12, 149.38)	0.17 (0.10, 0.28)	−5.24 (−5.36, −5.12)
East Asia	2118.96 (1755.79, 2532.71)	0.64 (0.52, 0.78)	93.34 (76.29, 116.63)	0.03 (0.03, 0.04)	−9.06 (−9.51, −8.62)
Eastern Europe	44.35 (43.18, 45.72)	0.09 (0.08, 0.09)	2.35 (2.16, 2.51)	0.01 (0.01, 0.01)	−8.10 (−8.74, −7.46)
Eastern Sub-Saharan Africa	662.13 (418.26, 952.56)	0.70 (0.44, 1.02)	275.02 (178.88, 433.77)	0.15 (0.10, 0.25)	−4.76 (−4.86, −4.65)
High-income Asia Pacific	8.81 (7.66, 10.14)	0.02 (0.02, 0.03)	1.00 (0.93, 1.09)	0.00 (0.00, 0.00)	−5.54 (−5.93, −5.16)
High-income North America	22.19 (21.54, 22.89)	0.04 (0.03, 0.04)	5.97 (5.41, 6.49)	0.01 (0.01, 0.01)	−4.00 (−4.55, −3.45)
North Africa and Middle East	3854.88 (2649.11, 5276.88)	2.72 (1.76, 3.83)	784.80 (576.34, 1112.06)	0.43 (0.31, 0.64)	−5.73 (−5.89, −5.58)
Oceania	92.14 (54.21, 135.66)	3.47 (1.94, 5.40)	136.21 (90.11, 196.92)	2.72 (1.69, 4.12)	−0.73 (−1.09, −0.37)
South Asia	10,457.83 (7480.77, 14,546.73)	2.40 (1.64, 3.39)	3496.39 (2710.85, 4474.48)	0.68 (0.50, 0.89)	−3.99 (−4.29, −3.69)
Southeast Asia	1758.27 (1366.44, 2170.03)	1.02 (0.70, 1.30)	519.71 (379.38, 636.76)	0.29 (0.21, 0.37)	−3.94 (−4.11, −3.76)
Southern Latin America	12.03 (10.80, 13.33)	0.08 (0.07, 0.09)	1.52 (1.30, 1.71)	0.01 (0.01, 0.01)	−6.53 (−7.00, −6.05)
Southern Sub-Saharan Africa	140.84 (108.48, 171.66)	0.68 (0.48, 0.89)	125.37 (97.35, 154.70)	0.51 (0.37, 0.66)	−0.91 (−1.79, −0.04)
Tropical Latin America	193.80 (175.92, 210.48)	0.35 (0.31, 0.38)	32.09 (26.88, 36.87)	0.06 (0.05, 0.07)	−5.15 (−5.83, −4.47)
Western Europe	28.99 (27.81, 30.36)	0.04 (0.04, 0.04)	7.72 (7.11, 8.35)	0.01 (0.01, 0.01)	−4.14 (−4.64, −3.65)
Western Sub-Saharan Africa	1553.03 (969.97, 2310.12)	1.62 (0.97, 2.44)	744.88 (418.53, 1043.30)	0.34 (0.19, 0.50)	−4.92 (−5.07, −4.77)

**Figure 4:**
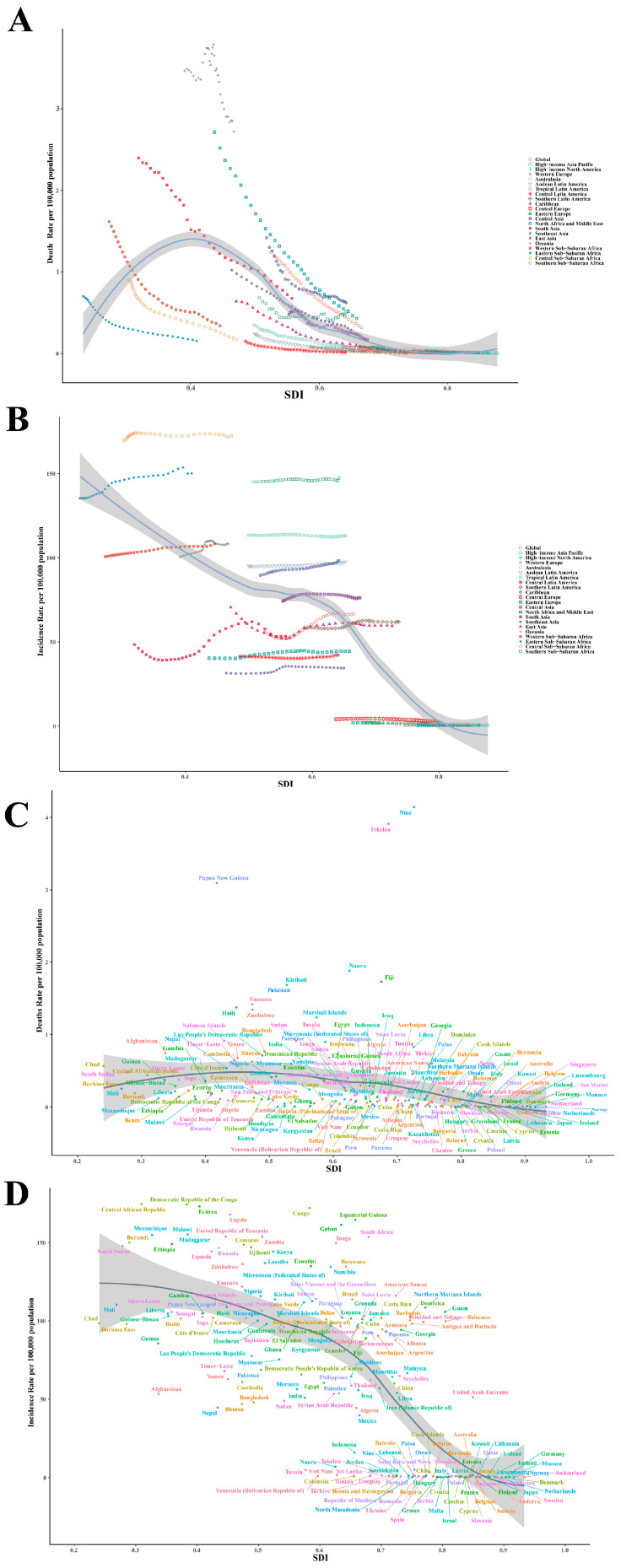
Association between SDI and pediatric RHD mortality (**A**) and incidence (**B**) rates by Global Burden of Disease regions, 1990–2021. Country-level association between SDI and pediatric RHD mortality (**C**) and incidence (**D**) rates in 2021.

The analysis of GBD datasets (1990–2021) examining ASDR patterns for RHD relative to SDI indices across 21 global territories (Figure 4A) uncovered a pronounced inverse relationship (Spearman’s r = −0.8110, 95% confidence interval: −0.8380 to −0.7803, *p* < 0.0001). Worldwide, death rates showed significant reduction, decreasing from 1.24 cases in 1990 to 0.32 cases per 100,000 by 2021, while persistent disparities emerged within high-SDI regions, which maintained consistently low rates, ranging below 0.1 per 100,000 and reaching 0.011 per 100,000 in Western Europe by 2021; middle-SDI regions like Southeast Asia achieved rapid reductions through healthcare improvements (1.02 to 0.29 per 100,000); and low-SDI regions sustained high burdens, with Oceania at 2.72 per 100,000 and Western Africa at 0.34 per 100,000 in 2021.

#### 3.4.2. DALYs

Between 1990 and 2021, there was a substantial decrease in the worldwide ASR of DALYs linked to rheumatic heart disease in children under 15 years old, indicating notable progress in reducing the disease burden in this age group (Table S1, Figure S4 in the Supplemental Material). Regionally, the most substantial declines in the ASRs of DALYs occurred in Eastern Europe (AAPC: −5.71%; 95% CI: −6.45 to −4.96) and North Africa and the Middle East (−5.09%; 95% CI: −5.20 to −4.97), while Oceania and Southern Sub-Saharan Africa exhibited more modest reductions (AAPC: −0.65% and −0.61%, respectively). Despite overall improvements, Oceania continued to report the highest ASR of DALYs in 2021, at 252.61 (95% UI: 166.05–371.38), followed by Central Sub-Saharan Africa (50.86; 95% UI: 30.70–77.64).

Analysis of age-adjusted DALY rates for RHD in relation to SDI indicators across 21 geographical zones during 1990–2021 demonstrated a pronounced inverse relationship. Statistical evaluation confirmed a robust negative association between these two variables throughout all examined regions (Spearman r = −0.8486, *p* < 0.001) (Figure S5 in the Supplemental Material). High-burden regions, including Oceania (252.61 per 100,000), Western Africa (52.58), and South Asia (68.82), exhibited persistently elevated burdens with low SDI (<0.5), while low-burden regions like High-income Asia Pacific (0.75) and Western Europe (1.24), with SDI > 0.8, showed minimal impact. East Asia achieved a 76% reduction, while the Caribbean presented an outlier with disproportionally high DALYs (73.69) despite moderate SDI, suggesting that non-economic factors play critical roles.

#### 3.4.3. Incidence

From 1990 through 2021, worldwide ASIR for RHD in the 0–14 age demographic exhibited a slight yet meaningful upward trend (Table 2, Figure 2D). Regionally, the highest ASIRs in 2021 were observed in Oceania (108.34, 95% UI: 62.27–166.66), The regions of Western Sub-Saharan Africa (108.28, with a 95% uncertainty interval ranging from 59.32 to 174.54) and Central Sub-Saharan Africa (172.43, with the 95% confidence limits falling between 94.78 and 279.76) exhibited notable variations, reflecting persistently elevated RHD incidence in low-SDI regions. Conversely, significant decreases were seen in High-income Asia Pacific (AAPC: −0.96%; 95% CI: −1.07 to −0.86), Central Europe (–1.30%; 95% CI: −1.39 to −1.21), and Western Europe (−0.78%; 95% CI: −1.01 to −0.55), suggesting effective control strategies in higher-SDI regions. Among GBD regions, East Asia reported a notable decrease in ASIR, from 70.63 (95% UI: 39.75–110.98) to 59.60 (95% UI: 33.29–94.08), with an AAPC of −0.50% (95% CI: −0.64 to −0.37).

**Table 2:** Age-standardized incidence rates (ASIR) for rheumatic heart disease among children aged 0–14 years; global numbers, and as categorized by region and SDI level, 1990–2021.

Location	1990 Cases	1990 ASIR (per 100,000, 95% UI)	2021 Cases	2021 ASIR (per 100,000, 95% UI)	AAPC (%, 95% CI)
Global	964,906.53 (612,864.83, 1,401,949.65)	55.84 (30.74, 89.59)	1,372,813.44 (855,165.80, 2,015,082.43)	66.76 (36.40, 107.60)	0.58 (0.51, 0.65)
High SDI	2999.24 (2280.29, 3800.35)	1.61 (1.07, 2.23)	2913.40 (2158.15, 3815.08)	1.68 (1.10, 2.34)	0.16 (0.06, 0.25)
High-middle SDI	77,049.13 (50,504.18, 110,713.83)	27.88 (16.05, 43.56)	57,333.11 (37,006.69, 83,380.04)	24.00 (13.61, 37.99)	−0.49 (−0.60, −0.37)
Middle SDI	378,595.58 (242,863.98, 551,449.88)	65.38 (36.20, 104.59)	368,120.29 (230,967.04, 538,617.54)	62.75 (34.64, 100.51)	−0.13 (−0.20, −0.06)
Low-middle SDI	290,038.70 (181,810.10, 422,916.15)	62.73 (33.84, 101.46)	432,380.50 (266,411.89, 638,413.08)	72.97 (39.22, 117.42)	0.47 (0.37, 0.57)
Low SDI	215,495.28 (135,914.11, 311,256.28)	99.21 (53.37, 159.11)	511,030.60 (319,158.12, 744,664.27)	112.14 (60.73, 180.56)	0.40 (0.36, 0.45)
Andean Latin America	14,067.94 (8792.05, 20,592.41)	95.16 (52.46, 154.03)	17,837.81 (11,138.45, 25,999.95)	97.57 (53.56, 156.43)	0.07 (0.05, 0.09)
Australasia	58.20 (36.42, 87.27)	1.28 (0.69, 2.11)	68.47 (44.75, 100.30)	1.22 (0.69, 1.95)	−0.13 (−0.18, −0.07)
Caribbean	10,133.55 (6372.12, 14,705.63)	89.56 (49.02, 143.98)	11,466.74 (7043.67, 16,614.70)	98.17 (53.04, 157.68)	0.30 (0.27, 0.32)
Central Asia	18,016.55 (11,650.95, 26,003.95)	74.28 (41.11, 119.02)	20,808.29 (13,221.22, 29,951.09)	76.19 (41.90, 121.97)	0.09 (0.07, 0.10)
Central Europe	1261.07 (876.60, 1730.24)	4.17 (2.51, 6.26)	507.48 (351.59, 689.23)	2.80 (1.70, 4.15)	−1.30 (−1.39, −1.21)
Central Latin America	26,536.81 (16,685.35, 38,481.34)	41.43 (23.08, 66.18)	27,795.24 (17,333.39, 40,694.21)	42.28 (23.37, 67.49)	0.06 (0.02, 0.10)
Central Sub-Saharan Africa	40,045.23 (26,112.58, 58,300.59)	169.56 (93.45, 270.70)	99,974.38 (63,171.12, 148,297.82)	172.43 (94.78, 279.76)	0.04 (0.02, 0.06)
East Asia	233,315.59 (152,549.16, 337,600.98)	70.63 (39.75, 110.98)	165,066.52 (106,616.69, 238,929.23)	59.60 (33.29, 94.08)	−0.50 (−0.64, −0.37)
Eastern Europe	999.73 (767.37, 1292.24)	1.93 (1.27, 2.73)	725.50 (535.18, 941.00)	2.00 (1.30, 2.85)	0.11 (0.06, 0.17)
Eastern Sub-Saharan Africa	116,098.77 (73,495.34, 166,583.75)	135.31 (73.33, 215.63)	265,831.73 (166,730.30, 387,394.78)	150.11 (80.85, 240.75)	0.34 (0.27, 0.40)
High-income Asia Pacific	196.04 (132.44, 291.71)	0.59 (0.34, 0.97)	87.44 (63.82, 118.99)	0.43 (0.27, 0.66)	−0.96 (−1.07, −0.86)
High-income North America	373.39 (259.06, 538.37)	0.60 (0.33, 0.98)	365.32 (263.59, 487.45)	0.57 (0.35, 0.88)	−0.10 (−0.25, 0.04)
North Africa and Middle East	56,012.11 (36,577.32, 80,344.35)	40.40 (22.44, 64.52)	82,627.50 (52,206.73, 120,413.64)	44.54 (24.00, 71.30)	0.33 (0.24, 0.42)
Oceania	2662.37 (1822.07, 3723.85)	100.48 (58.94, 154.22)	5421.94 (3657.68, 7504.45)	108.34 (62.27, 166.66)	0.24 (0.19, 0.30)
South Asia	206,098.45 (125,421.57, 303,887.98)	48.48 (25.34, 79.16)	283,144.94 (168,573.24, 424,659.77)	53.45 (27.90, 87.25)	0.33 (0.04, 0.63)
Southeast Asia	54,216.47 (34,649.23, 78,379.32)	31.53 (17.47, 50.30)	60,808.88 (38,560.46, 88,396.19)	34.46 (19.12, 55.20)	0.31 (0.26, 0.36)
Southern Latin America	8767.66 (5607.76, 12581.11)	58.42 (32.57, 92.74)	9401.80 (5899.63, 13,696.28)	61.97 (34.82, 98.49)	0.19 (0.17, 0.22)
Southern Sub-Saharan Africa	29,675.76 (18,789.85, 43,305.38)	145.15 (80.47, 233.94)	36,100.65 (22,949.18, 53,310.74)	147.30 (80.69, 237.49)	0.04 (0.01, 0.08)
Tropical Latin America	62,749.35 (39,463.29, 92,309.10)	113.44 (61.83, 181.79)	57,405.02 (36,170.54, 83,858.05)	113.20 (61.89, 181.94)	−0.01 (−0.01, −0.00)
Western Europe	295.88 (199.42, 435.06)	0.43 (0.23, 0.72)	209.32 (140.09, 304.04)	0.32 (0.17, 0.53)	−0.78 (−1.01, −0.55)
Western Sub-Saharan Africa	83,325.58 (53,319.15, 120,663.67)	100.69 (55.03, 162.25)	227,158.48 (143,526.47, 329,674.35)	108.28 (59.32, 174.54)	0.21 (0.17, 0.25)

Statistical evaluation using Spearman’s method demonstrated an inverse relationship of notable strength between socioeconomic development indicators and RHD prevalence (coefficient = −0.7295, 95% CI spanning −0.7706 to −0.6813, significance level *p* < 0.001), (Figure 4B). The observed inverse correlation reveals significant geographical variations, with areas of lower SDI—particularly Sub-Saharan Africa and South Asia—showing substantially elevated occurrence frequencies relative to more affluent regions, including Western Europe and North America, highlighting inequities in global disease distribution and necessitating targeted interventions in low-resource settings. Temporal trends from 1990 to 2021 reinforced this pattern, with high-SDI regions demonstrating sustained declines in incidence, while low-SDI regions maintained elevated rates.

#### 3.4.4. Prevalence

Between 1990 and 2021, the prevalence of RHD in pediatric populations (0–14 years) exhibited a slight yet statistically meaningful increase worldwide in age-standardized prevalence rates (ASPR) (Table S2, Figure S6 in Supplemental Material). Among GBD regions, Central Sub-Saharan Africa recorded the highest ASPR in 2021, at 751.30 (95% UI: 485.77–1094.51), while Western Europe had the lowest, at 3.88 (95% UI: 2.70–5.38). The most pronounced declines were seen in Central Europe (AAPC: −0.89%; 95% CI: −0.95 to −0.83) and Western Europe (AAPC: −0.53%; 95% CI: −0.73 to −0.34). Conversely, in several low- and middle-income regions, notable rises in ASPR were observed across multiple regions, particularly in Sub-Saharan Africa’s eastern and western zones, as well as in the Pacific island territories, with these increases demonstrating statistical significance, underscoring persistent inequalities in RHD burden.

The graphical representation demonstrates the fluctuating correlations between age-adjusted RHD occurrence levels and SDI values, spanning 21 geographical areas over a 31-year period from 1990 onward, revealing a significant negative correlation (r = −0.7274, *p* < 0.001) with higher RHD prevalence in low-SDI regions (Figure S7 in the Supplemental Material). Oceania exhibited the highest prevalence, at 572.61 per 100,000 in 2021, while Western Europe recorded the lowest, at 3.88 per 100,000. High-SDI regions achieved steady declines through antibiotic stewardship, whereas low-SDI regions maintained persistent high burdens, with Central Sub-Saharan Africa reaching 751.30 per 100,000, underscoring systemic gaps. South Asia presented as an outlier, with a rising prevalence peaking at 273.47 per 100,000 in 2015.

### 3.5. National Variation

#### 3.5.1. Mortality

**Figure 5:**
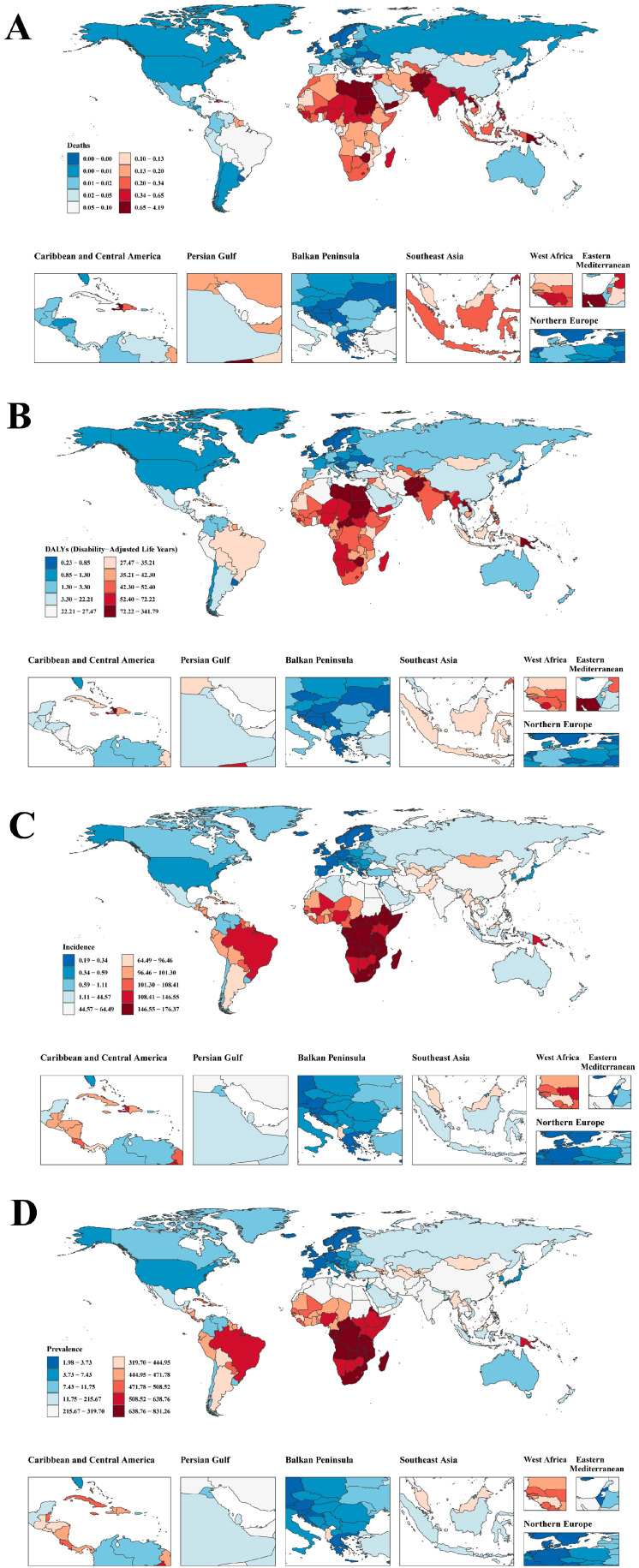
Global distribution of age-standardized rate of (**A**) death, (**B**) disability-adjusted life years (DALYs), (**C**) incidence, and (**D**) prevalence associated with rheumatic heart disease among children aged 0–14 years, 2021.

In 2021, the worldwide ASDR attributed to RHD in children aged 0–14 showed significant geographical disparities (Figure 5A). Developing nations demonstrated substantially elevated mortality rates, with the Western Pacific region and Sub-Saharan Africa being particularly affected. Niue reported the most severe ASDR figures, which reached 4.15 per population unit (95% UI: 2.69–5.88), followed by Papua New Guinea at 3.09 (95% UI: 1.83–4.80), and Tokelau at 3.91 (95% UI: 2.49–5.83). In contrast, many high-income countries, such as Sweden (0.001; 95% UI: 0.0008–0.0013), Norway (0.0015; 95% UI: 0.0012–0.0018), and the United Kingdom (0.0037; 95% UI: 0.0033–0.0041), recorded the lowest ASMR values.

This study also analyzed age-standardized RHD mortality rates across 204 countries using 2021 GBD data, demonstrating a strong inverse relationship with SDI (r = −0.7204, *p* < 0.001) (Figure 4C). High-burden countries like Pakistan, Niger, and Zimbabwe exceeded 1.5 per 100,000, surpassing 150-fold the rates of low-burden nations such as Sweden and Norway (<0.01 per 100,000). Outliers emphasized SDI’s pivotal role: Niue recorded the highest mortality (4.15 per 100,000) with the lowest SDI, while Sweden had the lowest mortality (0.001 per 100,000) with the highest SDI, highlighting urgent needs for streptococcal infection control and healthcare strengthening in low-SDI settings.

#### 3.5.2. DALYs

In 2021, the global ASRs of DALYs due to RHD among children aged 0–14 years showed striking disparities across countries and regions (Figure 5B). The greatest disease burden was concentrated in developing nations, with Oceania and parts of Africa showing particularly severe impacts on population health. Papua New Guinea reported the greatest burden, at 283.41 (95% UI: 178.84–427.86), followed by Tokelau (321.18; 95% UI: 205.60–477.40), and Niue (338.40; 95% UI: 220.59–479.15). Conversely, the lowest rates were documented in high-income countries such as Sweden (0.23; 95% UI: 0.17–0.31), Norway (0.28; 95% UI: 0.21–0.37), and the United Kingdom (0.57; 95% UI: 0.46–0.71).

This study also analyzed the age-standardized DALYs rates of RHD across 204 countries in 2021 using the GBD data, revealing a strong inverse correlation between SDI and RHD burden (Spearman’s r = −0.7880, *p* < 0.001) (Figure S8 in the Supplemental Material). Low-SDI regions like Pakistan, Niger, and Papua New Guinea showed the highest rates, exceeding 100 per 100,000, while high-SDI nations like Norway and Sweden had minimal rates, ranging below 1 per 100,000. Pacific Island nations and sub-Saharan African countries demonstrated exceptionally high burdens, reflecting healthcare access barriers. Moderate-SDI countries, including China (17.07) and Brazil (29.93), exceeded predicted values, suggesting delayed public health interventions during economic transition.

#### 3.5.3. Incidence

The worldwide incidence of RHD in children aged 0–14 during 2021 exhibited significant regional variations (Figure 5C). Sub-Saharan African countries recorded the highest ASIRs, exceeding 150 per 100,000 in the Democratic Republic of Congo (174.35; 95% UI: 95.73–281.84), Central African Republic (174.63; 95% UI: 94.96–279.34), and Eritrea (173.10; 95% UI: 92.93–281.04). Pacific Island nations also demonstrated elevated rates, including Tonga (149.79; 95% UI: 86.48–231.83), Vanuatu (121.92; 95% UI: 69.71–185.66), and Samoa (114.20; 95% UI: 66.08–174.20). Conversely, Western European and East Asian countries maintained minimal incidence rates: Sweden (0.19; 95% UI: 0.09–0.32), Japan (0.42; 95% UI: 0.29–0.61), and the United Kingdom (0.34; 95% UI: 0.18–0.55).

The research further examined the relationship between age-adjusted RHD incidence rates and SDI values among 204 nations during 2021, revealing a strong negative correlation (Spearman’s r = −0.7825, *p* < 0.001) (Figure 4D). Geographic disparities were stark. The areas characterized by elevated socioeconomic development indices, particularly Western Europe and North America, demonstrated remarkably low rheumatic heart disease incidence rates, consistently recording fewer than one case per 100,000 individuals (Sweden: 0.19, Denmark: 0.20), whereas low-SDI regions, including sub-Saharan Africa and Oceania, reported rates exceeding 100 per 100,000 (Central African Republic: 174.63, Tonga: 149.79).

#### 3.5.4. Prevalence

The 2021 data revealed significant regional variations in the ASPRs of RHD for children aged 0–14, with pronounced differences in disease prevalence between nations (Figure 5D). The highest ASPRs were recorded in Sub-Saharan Africa and in Pacific Island nations, with Tonga reporting the highest rate, at 823.03 (95% UI: 571.18–1149.92), followed closely by Vanuatu at 651.26 (95% UI: 443.93–906.73), and Samoa at 634.68 (95% UI: 437.13–891.09). In contrast, the lowest prevalence rates were found in Western Europe and high-income Asian countries such as Sweden (2.00; 95% UI: 1.31–2.78), Denmark (2.39; 95% UI: 1.31–3.73), and Japan (6.14; 95% UI: 4.89–7.64).The analyzed ASPRs of RHD across 204 countries in 2021, using GBD data, revealed a strong negative correlation between SDI and RHD prevalence (Spearman’s r = −0.7426, *p* < 0.001) (Figure S9 in the Supplemental Material). The scatterplot demonstrated an inverse gradient, with highest prevalence in low-SDI regions like Sub-Saharan Africa and Oceania, and the lowest in high-SDI nations. The Central African Republic (SDI: 0.29) had 749.8 per 100,000, while Sweden (SDI: 0.92) reported only 2.0 per 100,000. High-burden areas included Oceania, with Tonga at 823.0 per 100,000, while high-SDI countries consistently showed a prevalence below 5 per 100,000, reflecting effective prevention strategies.

#### 3.5.5. AAPC Changes

**Figure 6:**
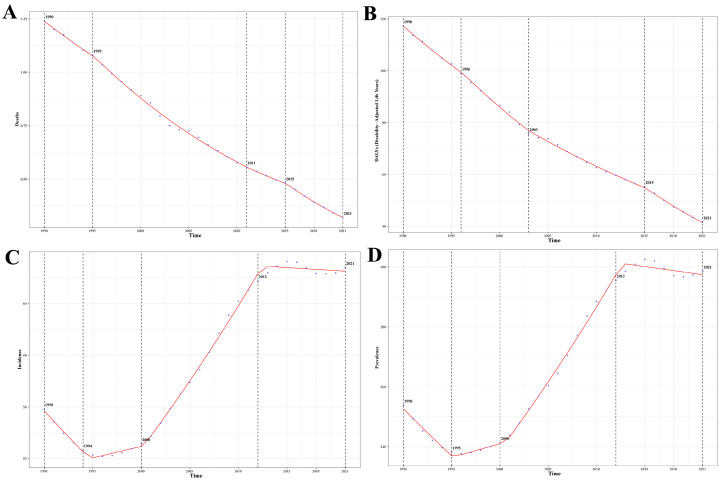
Global average annual percent change (AAPC) in age-standardized (**A**) death, (**B**) DALY, (**C**) incidence, and (**D**) prevalence rates associated with rheumatic heart disease in children aged 0–14 years, 1990–2021.

From 1990 to 2021, the worldwide impact of RHD in pediatric populations aged 0–14 years, evaluated through ASDR and composite health loss indicators (i.e., DALY rates), showed a consistent and statistically significant downward trend, with varying degrees of reduction across different periods (Figure 6A,B). The ASDR declined, with an AAPC of −2.77% (95% CI: −3.26 to −2.27) from 1990 to 1995, and accelerated to −4.07% (95% CI: −4.18 to −3.96) during 1995–2011, and further to –6.67% (95% CI: −7.15 to −6.20) between 2015 and 2021, all with *p* < 0.001, indicating high statistical significance. A similar decreasing pattern was observed in DALY rates, with AAPCs of −2.73% (95% CI: −3.00 to −2.45) from 1990 to 1996, −3.64% (95% CI: −3.92 to −3.37) from 1996 to 2003, –2.77% (95% CI: −2.89 to −2.65) from 2003 to 2015, and a notably steeper decline of –4.66% (95% CI: −5.00 to −4.31) from 2015 to 2021; all were statistically significant at *p* < 0.001.

Between 1990 and 2021, the worldwide ASIR and ASPR for RHD in children under 15 exhibited variable trends with intermittent rises and declines, with distinct phases of increase and decline (Figure 6C,D). For ASIR, between 1990 and 1994, a notable downward trend was observed, marked by an annual percentage decrease of 1.46% (95% CI: −1.74 to −1.19; statistical significance *p* < 0.001). Subsequently, the years 1994 through 2000 witnessed a marginal upward shift, registering a growth rate of 0.35% per annum (95% CI: 0.13 to 0.56; *p*-value = 0.0015), and there was a more pronounced rise from 2000 to 2012 at 1.92% (95% CI: 1.85 to 2.00; *p* < 0.001). However, the trend plateaued during 2012–2021, with a nonsignificant APC of −0.07% (95% CI: −0.18 to 0.05; *p* = 0.242). A similar pattern was observed in ASPR: a significant decline from 1990 to 1995 at −1.28% (95% CI: −1.51 to −1.05; *p* < 0.001), followed by increases from 1995 to 2000 (0.39%; 95% CI: 0.08 to 0.70; *p* = 0.014) and in 2000–2012 (1.84%; 95% CI: 1.76 to 1.93; *p* < 0.001). Like incidence, prevalence rates also stabilized between 2012 and 2021 with a mild but statistically significant decline of −0.15% (95% CI: −0.28 to −0.02; *p* = 0.019).

#### 3.5.6. APC Analysis

**Figure 7:**
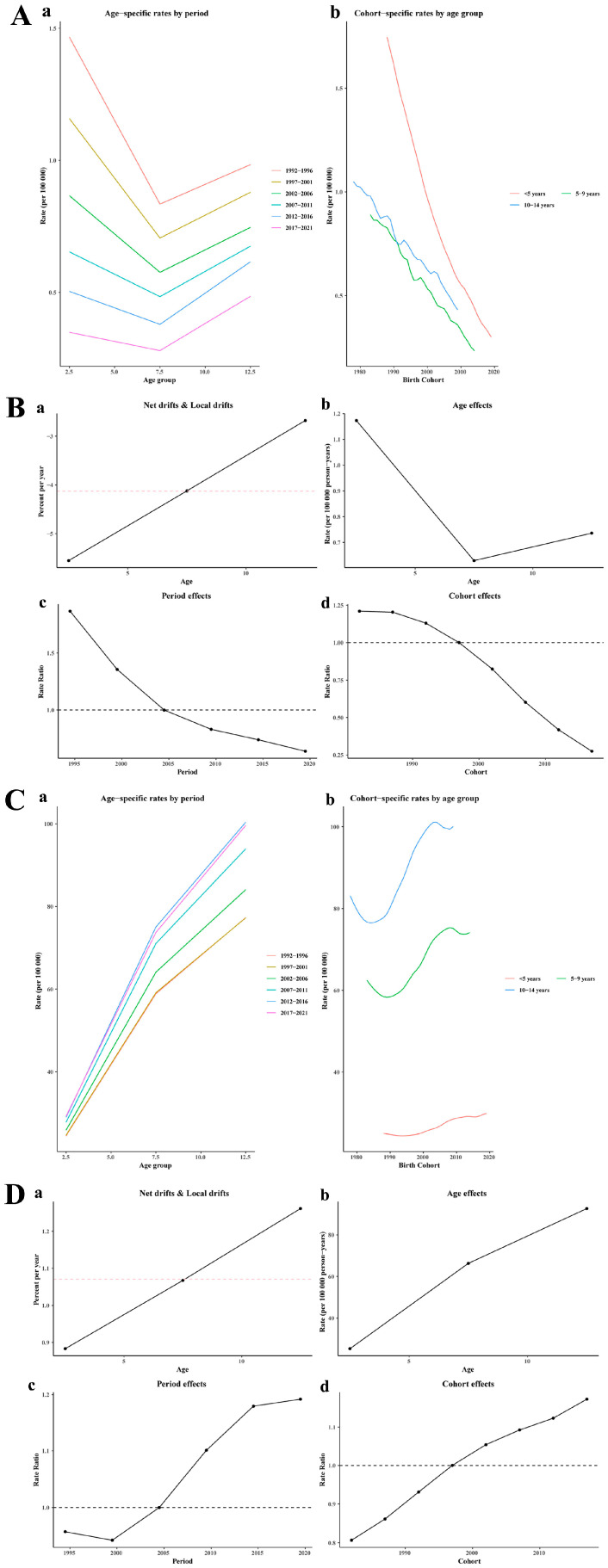
Global age–period–cohort analysis model results for rheumatic heart disease death (**A**) and incidence (**C**) among children aged 0–14 years; global, 1990–2021 (a, Age-specific rates by period; b Cohort-specific rates by age group). Global net-drift results for rheumatic heart disease death (**B**) and incidence (**D**) among children aged 0–14 years; global, 1990–2021 (a, Net drifts & Local drifts, b, Age effects; c, Period effects; d, Cohort effects). The pink horizontal dashed line represents the net drift, which is the overall annual trend (a), The black horizontal dashed line at 1.0 represents the reference level (c,d).

The APC analysis showed contrasting trends globally in RHD mortality and incidence among children aged 0–14 years, extending from 1990 to 2021 (Figure 7A–D). A significant downward trend was observed, with an overall net drift of –4.13% (95% CI: −4.22 to −4.04; *p* < 0.001). The local drift showed age-specific declines, which were steepest in children under 5 years (−5.55%; 95% UI: −5.69 to −5.42), followed by those 5–9 years old (–4.12%; 95% UI: −4.23 to −4.01), and smallest within the ages of 10 to 14 years (–2.68%; 95% UI: −2.81 to −2.55). Period effects declined from 1.87 (1994.5) to 0.64 (2019.5), while cohort effects decreased from 1.21 (1982) to 0.27 (2017), confirming generational survival improvements. In contrast with the mortality trends, incidence showed significant upward trends, with a net drift of +1.07% annually (95% CI: 1.02–1.13; *p* < 0.001). Local drifts ranged from +0.88% (age: 2.5 years) to +1.26% (age: 12.5 years). Age effects increased from 25.29 per 100,000 in children <5 years to 92.64 at 10–14 years. Period and cohort effects both rose consistently through 2019 and 2017, respectively. All APC components showed statistical significance (*p* < 0.001).

#### 3.5.7. Inequality Analysis

**Figure 8:**
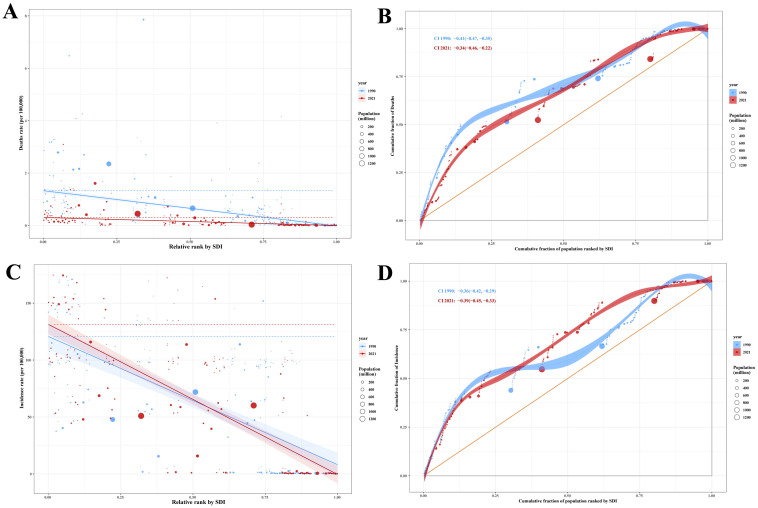
Slope and concentration indices of inequality for RHD-related deaths (**A**,**B**) and incidence (**C**,**D**) in children aged 0–14 years, by SDI ranking, 1990 vs. 2021.

From 1990 to 2021, disparities in childhood RHD health outcomes globally exhibited inconsistent patterns when assessed through the SII and the CII, as illustrated in Figure 8A through Figure 8D. The analysis revealed fluctuating gradients rather than uniform trends across different socioeconomic strata. DALYs and mortality inequalities narrowed substantially. DALY-related SII improved from −143.87 (95% UI: −160.49 to −127.24) in 1990 to −57.76 (95% UI: −64.10 to −51.41) in 2021, with CII improving from −0.40 to −0.35. Similarly, mortality SII decreased from −1.35 (95% UI: −1.51 to −1.18) to −0.31 (95% UI: −0.36 to −0.25), while CII improved from −0.41 to −0.34, indicating reduced absolute and relative gaps between lowest and highest SDI quintiles. Incidence and prevalence inequalities have intensified. Incidence SII worsened from –112.60 (95% UI: −131.23 to −93.97) to −131.90 (95% UI: −147.77 to −116.03), with CII declining from −0.36 to −0.39. Prevalence inequality similarly deteriorated: SII dropped from −495.54 (95% UI: −580.80 to −410.27) to −587.19 (95% UI: −660.97 to −513.42), while CII declined from −0.33 to −0.37, demonstrating an increased concentration of disease burden in socioeconomically disadvantaged regions (Figures S10–S13 in the Supplemental Material).

## 4. Discussion

This research conducted a comprehensive analysis to measure the worldwide, local, and country-specific impacts of RHD in children, considering a time period between 1990 and 2021, and employing datasets from the GBD 2021 investigation. The methodology involved detailed statistical evaluations across different geographical scales, aiming to determine disease prevalence trends over this three-decade period. Our primary results reveal a nuanced epidemiological landscape. While the global ASDR among children declined significantly, from 1.24 to 0.32 per 100,000 (AAPC: −4.27%), this positive trajectory was not uniformly shared across all socio-demographic regions. High-SDI countries achieved the largest improvements in mortality, likely reflecting the synergistic benefits of effective prevention, early detection, and robust healthcare infrastructure. Conversely, low- and low-middle-SDI regions still bore excessively high proportions of the RHD burden, with slower reductions in death rates and persistently high DALYs.

Contrary to mortality trends, the global burden of RHD incidence and prevalence increased modestly over the same period. The ASIR rose from 55.84 to 66.76 per 100,000 (AAPC: 0.58%), and the prevalence rate (ASPR) climbed from 253.82 to 298.55 per 100,000 (AAPC: 0.53%). These trends likely reflect a combination of improved diagnostic sensitivity, population growth, and limited control of primary streptococcal infections in endemic regions. Importantly, APC analysis indicated significant generational improvements in mortality, with the steepest declines being observed in younger age groups, especially in children under 5 years.

Disparities according to level of socioeconomic development, as captured by the SDI, were stark and persistent. RHD incidence, prevalence, and mortality all showed strong inverse associations with SDI levels. Nations with limited socioeconomic development indicators, particularly those in regions like Sub-Saharan Africa and South Asia, demonstrated the most elevated age-standardized rates in every measurement category, reinforcing the disease’s well-established link to poverty, overcrowding, and inadequate access to healthcare. Though mortality has improved even in these regions, the pace lags that of high-income settings, suggesting that upstream determinants, such as rheumatic fever prevention, timely penicillin prophylaxis, and surgical intervention, remain under-addressed.

The trends identified in this study, declining mortality and DALYs, when contrasted with rising incidence and prevalence, can reveal multifactorial mechanisms involving both biomedical and systemic components. At the biological level, the pathogenesis of RHD originates from repeated episodes of acute rheumatic fever, an autoimmune sequela of untreated GAS pharyngitis in genetically susceptible children [22]. In regions where streptococcal infections remain endemic and inadequately managed, the cycle of reinfection and immune-mediated valvular injury perpetuates the high incidence of disease. The consistent reduction in RHD-related deaths and DALYs, particularly in high-SDI regions, is likely driven by improved access to timely diagnosis and effective secondary prevention strategies. Expanded echocardiographic screening has played a crucial role in early detection of subclinical or latent RHD, allowing initiation of secondary prophylaxis and thereby preventing disease progression [23].

Notably, the decline in mortality in younger children may be influenced by broad improvements in child health services, including better antenatal care, expanded vaccination coverage, and reductions in comorbidities such as malnutrition and respiratory infections that previously had worsened RHD outcomes [8,24,25,26]. However, these improvements have not been universally shared. In low-SDI regions, barriers such as inconsistent access to healthcare, lack of trained providers, and weak surveillance systems contribute to persistent high RHD burden and later-stage diagnosis [8]. The stagnation or even increase in incidence in several regions raises an urgent call to refocus on primary prevention, particularly the timely diagnosis and treatment of streptococcal pharyngitis. Evidence supports the finding that school-based screening and treatment of sore throats can drastically reduce ARF incidence when combined with health education and antibiotic accessibility [10]. Equally important is the scale-up of echocardiographic screening programs, which have been shown to detect early and asymptomatic cases, allowing earlier initiation of prophylaxis and reductions in long-term sequelae [3,6].

The APC effects observed in our analysis reinforce the notion of generational gains in RHD control. The marked reduction in burden among more recent birth cohorts, particularly in high-SDI countries, suggests that long-standing investments in maternal–child health systems, school-based screening, and antibiotic accessibility are beginning to yield dividends [8]. Nonetheless, stagnation or slower gains in low-income countries may reflect systemic barriers, such as inadequate health infrastructure, poor sanitation, and limited health education, that continue to impede disease control. The current study identifies a global increase in incidence and prevalence from 1990 to 2021; prior meta-analyses have reported a plateau or even a decline in RHD prevalence, particularly in school-based cohorts from high-burden regions such as Sub-Saharan Africa [3,27]. This discrepancy can be attributed to differences in the study design, data aggregation methods, and scope of age groups analyzed. The apparent rise in prevalence in this study is consistent with expanded use of the World Heart Federation criteria, which identify early or mild cases previously classified as normal under older WHO criteria [23].

This research demonstrates notable robustness through its extensive examination of worldwide, regional, and country-specific patterns in childhood RHD prevalence spanning three decades. Unlike previous single-site or age-limited reports [28,29], this examination utilizes normalized metrics derived from the 2021 Global Disease Impact assessment protocol, ensuring internal consistency and broad comparability. By focusing on the 0–14 years age group, it addresses a critical gap in the prior literature and isolates pediatric dynamics of disease onset and progression. The inclusion of detailed estimates for incidence, prevalence, mortality, and DALYs allows for nuanced interpretation across socio-demographic strata. The use of advanced methods—such as age–period–cohort modeling and AAPC—provides granular insights into temporal shifts. These analytical approaches strengthen the reliability of trends and offer valuable indicators for evaluating public health interventions and policies globally.

Despite its strengths, this study has several limitations. First, as with all GBD analyses, the estimates rely heavily on modeling techniques where empirical data are sparse. This is particularly relevant in low-income countries, where vital registration and echocardiographic screening are limited, possibly leading to underestimation or misclassification. Second, while age-standardized indicators improve comparability, they may obscure critical within-country disparities across socioeconomic and ethnic groups. Third, the GBD study aggregates all RHD-related outcomes without distinguishing between definite and borderline cases, which could influence prevalence trends. Finally, although APC modeling offers insights into temporal shifts, it is sensitive to assumptions and may not fully capture real-time disruptions, such as those caused by migration or pandemic-related healthcare access interruptions. These methodological constraints suggest that while the trends are robust at the population level, localized verification remains essential for policy translation.

## 5. Conclusions and Perspectives

This research provides an extensive analysis of childhood RHD prevalence worldwide between 1990 and 2021, highlighting both significant achievements and persistent challenges. The marked declines in age-standardized mortality and DALYs reflect meaningful progress in secondary prevention and healthcare delivery. However, the concurrent rises in incidence and prevalence, especially in low-SDI regions, underscore the urgent need to strengthen primary prevention and address the upstream determinants of the disease.

To accelerate progress, national health systems should integrate RHD control into maternal and child health programs, expand echocardiographic screening, and improve access to antibiotics for streptococcal infections. International collaboration, regional surveillance platforms, and community-engaged interventions are essential to eliminate RHD as a public health concern. Achieving equity in disease outcomes will require not only clinical solutions but also structural investments targeting poverty, overcrowding, and health system fragility.

## Data Availability

The original contributions presented in this study are included in the article and Supplementary Material. Further inquiries can be directed to the corresponding author.

## Supplementary Materials

The following supporting information can be downloaded at: https://www.mdpi.com/article/10.3390/children12070843/s1. Table S1: Age-standardized disability-adjusted life year rates of rheumatic heart disease among children aged 0–14 years, 1990–2021; Table S2: Age-standardized prevalence rate (ASPR) of rheumatic heart disease among children aged 0–14 years by global, regional, and SDI-level, 1990–2021; Figure S1: Analytical framework and data flow for assessing pediatric rheumatic heart disease burden from 1990 to 2021; Figure S2: Age-standardized DALY rates of rheumatic heart disease among children aged 0–14 years by sex and region, 2021; Figure S3: Age-standardized prevalence rates (ASPR) of rheumatic heart disease among children aged 0–14 years by sex and region, 2021; Figure S4: Average annual percentage change (AAPC) in age-standardized disability-adjusted life years (DALYs) rates due to rheumatic heart disease among children aged 0–14 years by region, 1990–2021; Figure S5: Average annual percentage change (AAPC) in age-standardized prevalence rates due to rheumatic heart disease among children aged 0–14 years by region, 1990–2021; Figure S6: Association between SDI and pediatric RHD DALYs rates by GBD regions, 1990-2021; Figure S7: Association between SDI and pediatric RHD prevalence rates by GBD regions, 1990-2021; Figure S8: Country-level association between SDI and pediatric RHD DALYs rates in 2021; Figure S9: Country-level association between SDI and pediatric RHD prevalence rates in 2021; Figure S10: Slope indices of inequality for RHD-related DALYs in children aged 0–14 years, by SDI ranking, 1990 vs. 2021; Figure S11: Concentration indices of inequality for RHD-related DALYs in children aged 0–14 years, by SDI ranking, 1990 vs. 2021; Figure S12: Slope indices of inequality for RHD-related prevalence, in children aged 0–14 years, by SDI ranking, 1990 vs. 2021; Figure S13: Concentration indices of inequality for RHD-related prevalence in children aged 0–14 years, by SDI ranking, 1990 vs. 2021.
