# Supplementary material for: Global Burden of Pediatric Rheumatic Heart Disease, 1990–2021: Analysis of the GBD 2021 Study
Source: Children (Basel). 2025 Jun 26;12(7):843. doi: 10.3390/children12070843 (PMC12293350; doi:10.3390/children12070843)
Supplement: Supplementary file 1 [file children-12-00843-s001.zip › Supplemental Figure.pdf]

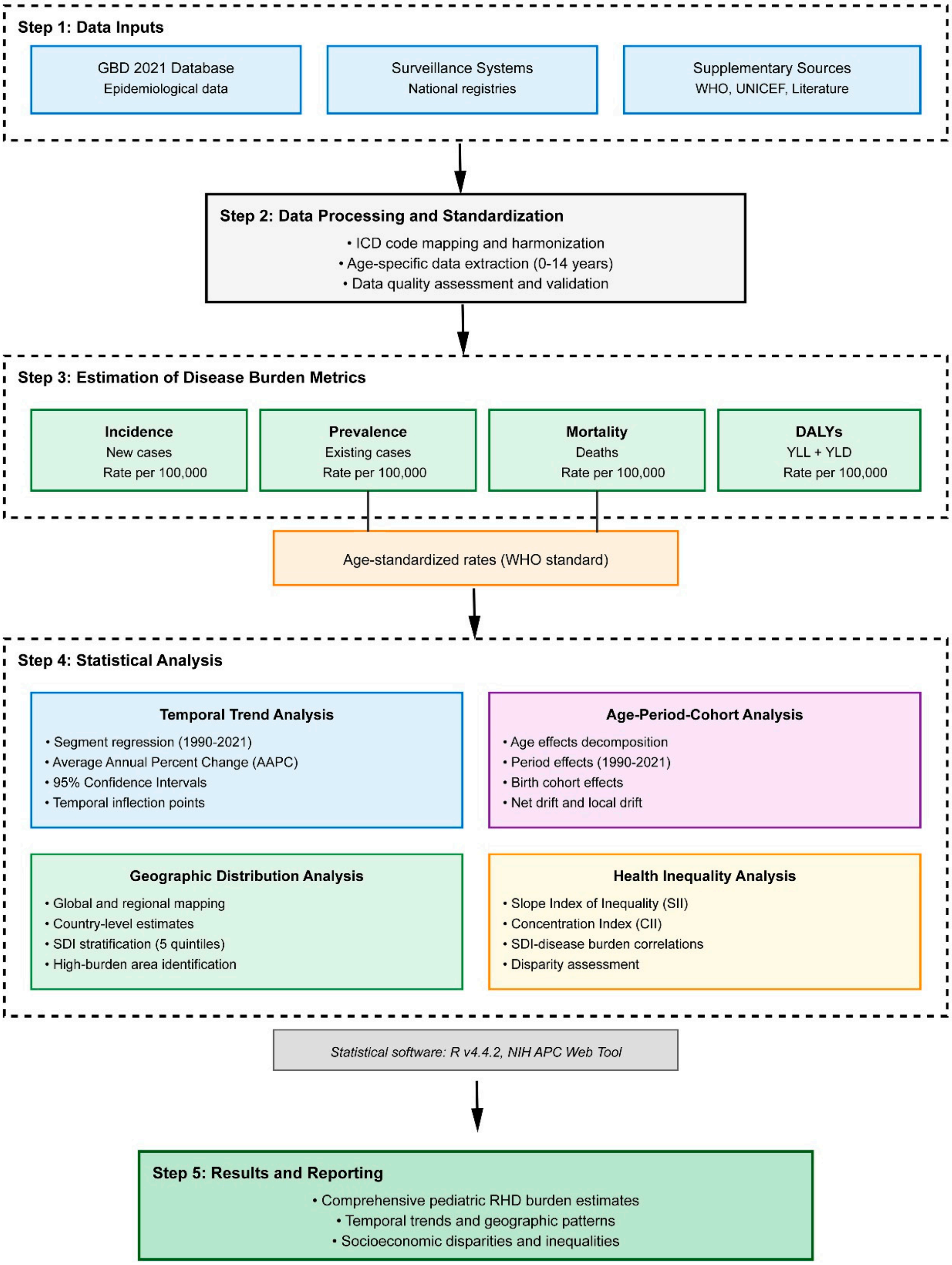

Figure S1 Analytical framework and data flow for assessing pediatric rheumatic heart disease burden from 1990 to 2021

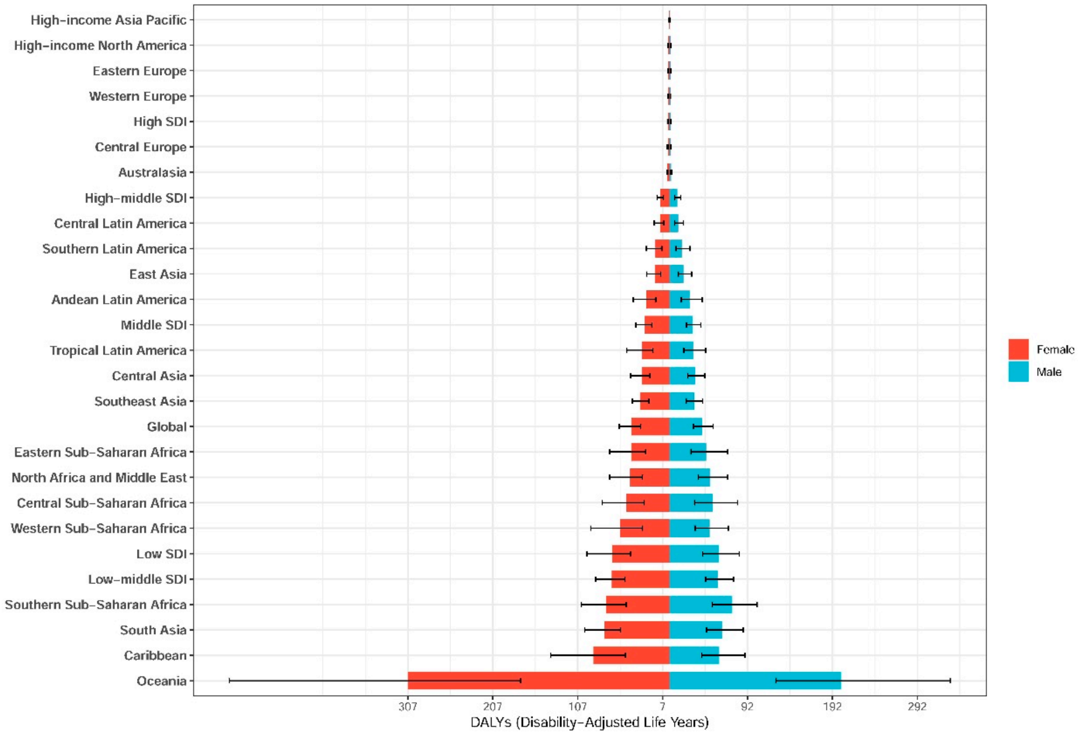

Figure S2 Age-standardized DALY rates of rheumatic heart disease among children aged 0–14 years by sex and region, 2021

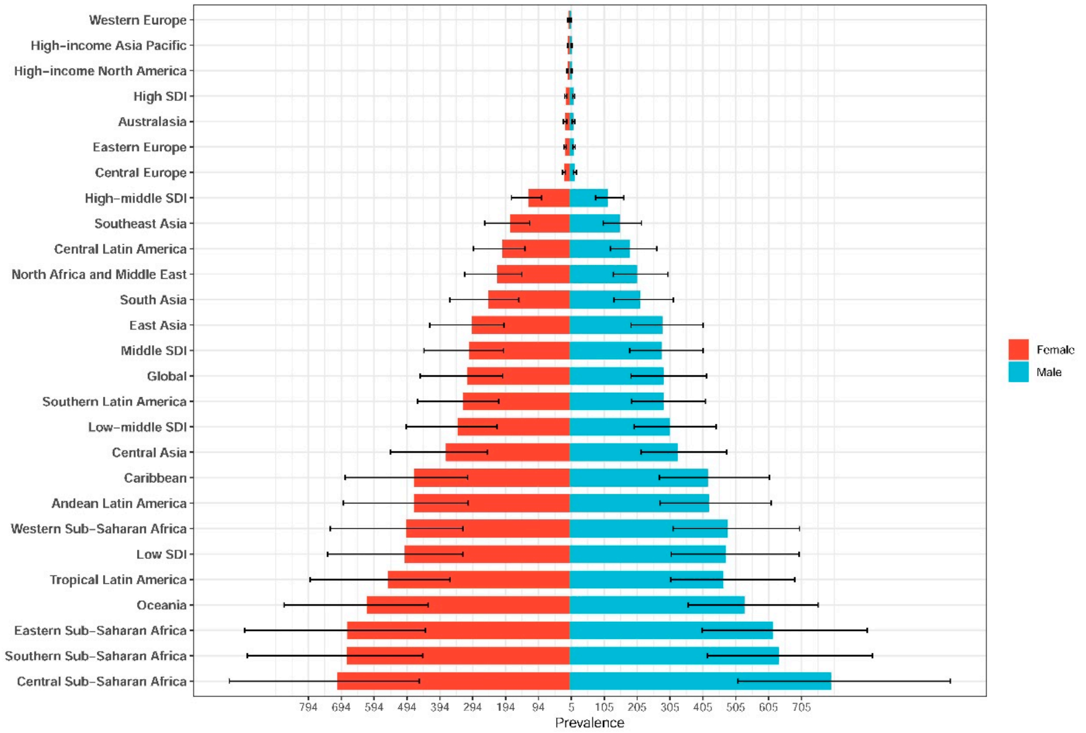

Figure S3 Age-standardized prevalence rates (ASPR) of rheumatic heart disease among children aged 0–14 years by sex and region, 2021

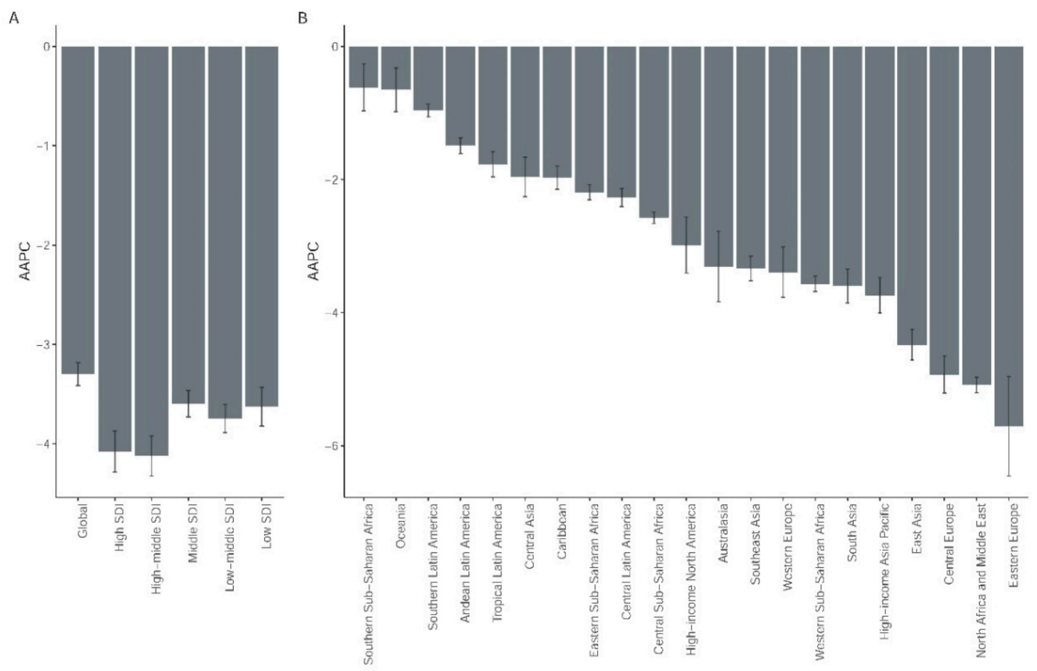

Figure S4 Average annual percentage change (AAPC) in age-standardized disability-adjusted life years (DALYs) rates due to rheumatic heart disease among children aged 0–14 years by region, 1990–2021

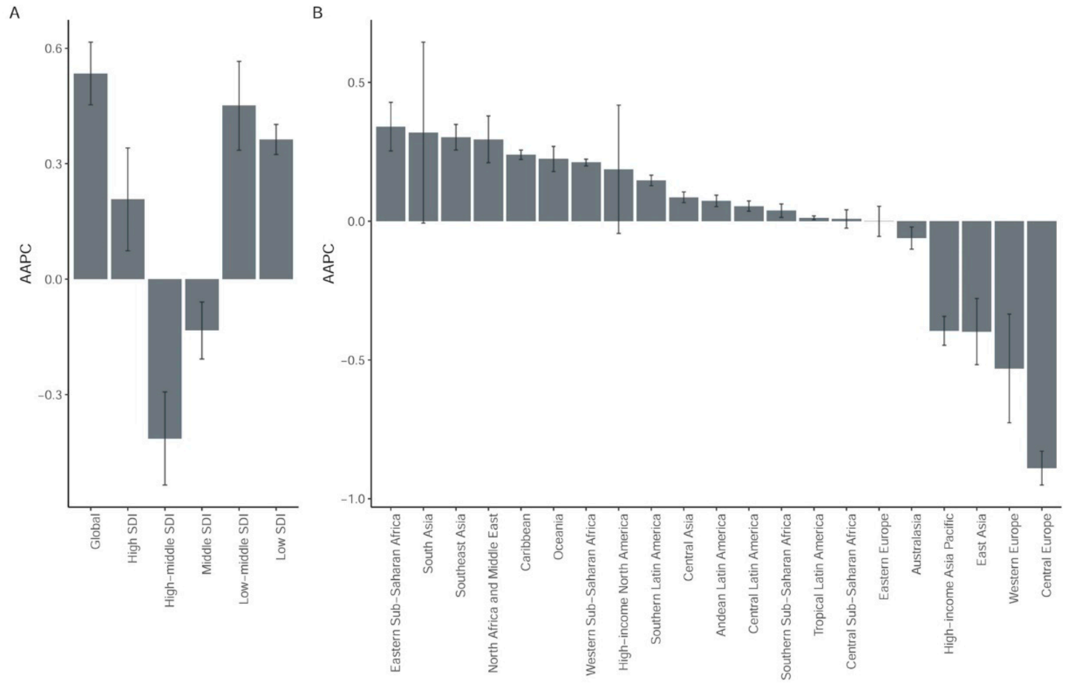

Figure S5 Average annual percentage change (AAPC) in age-standardized prevalence rates due to rheumatic heart disease among children aged 0–14 years by region, 1990–2021

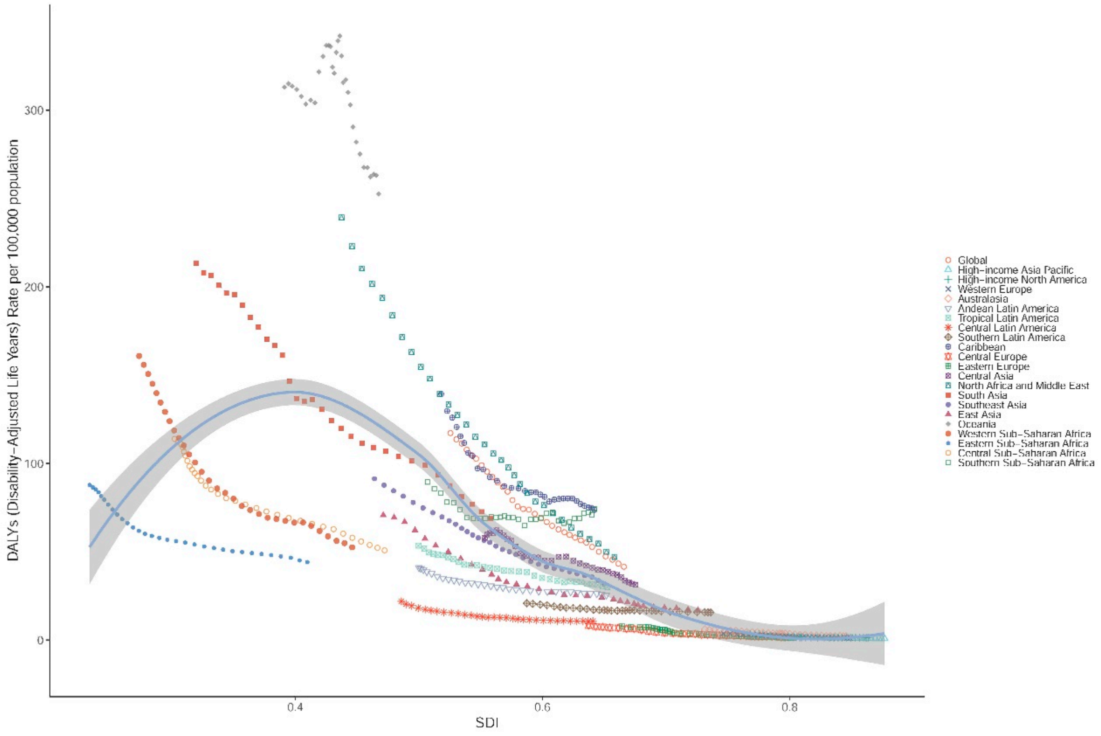

Figure S6 Association between SDI and pediatric RHD DALYs rates by GBD regions, 1990-2021.

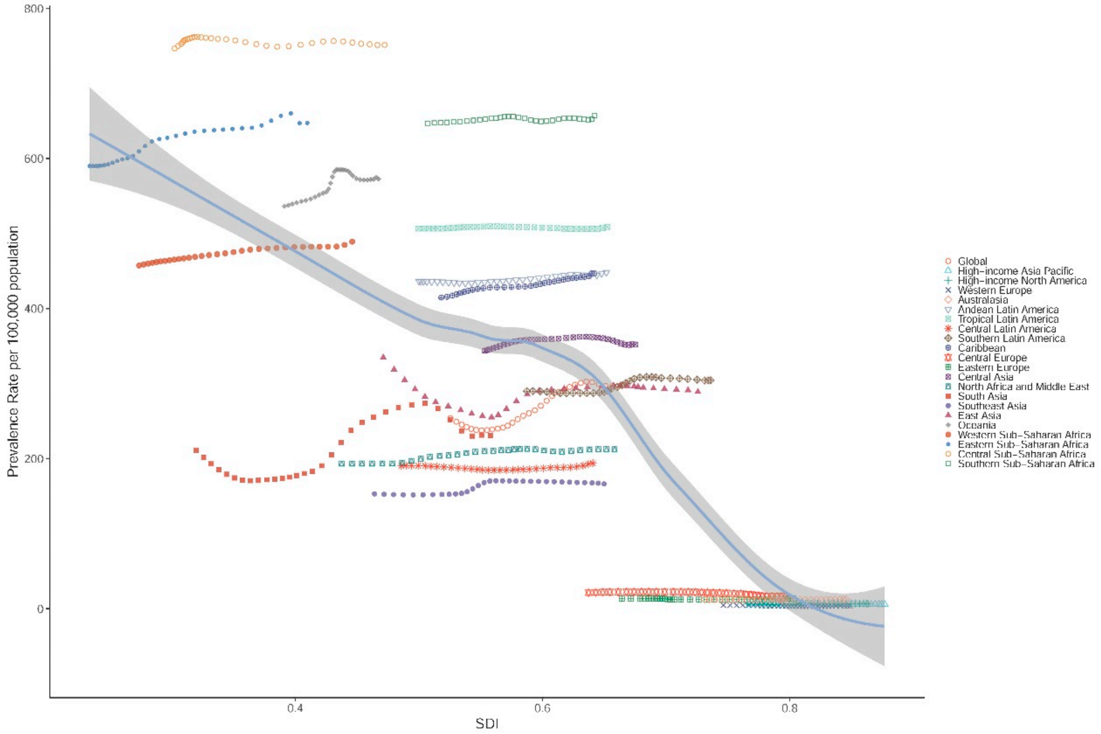

Figure S7 Association between SDI and pediatric RHD prevalence rates by GBD regions, 1990-2021.

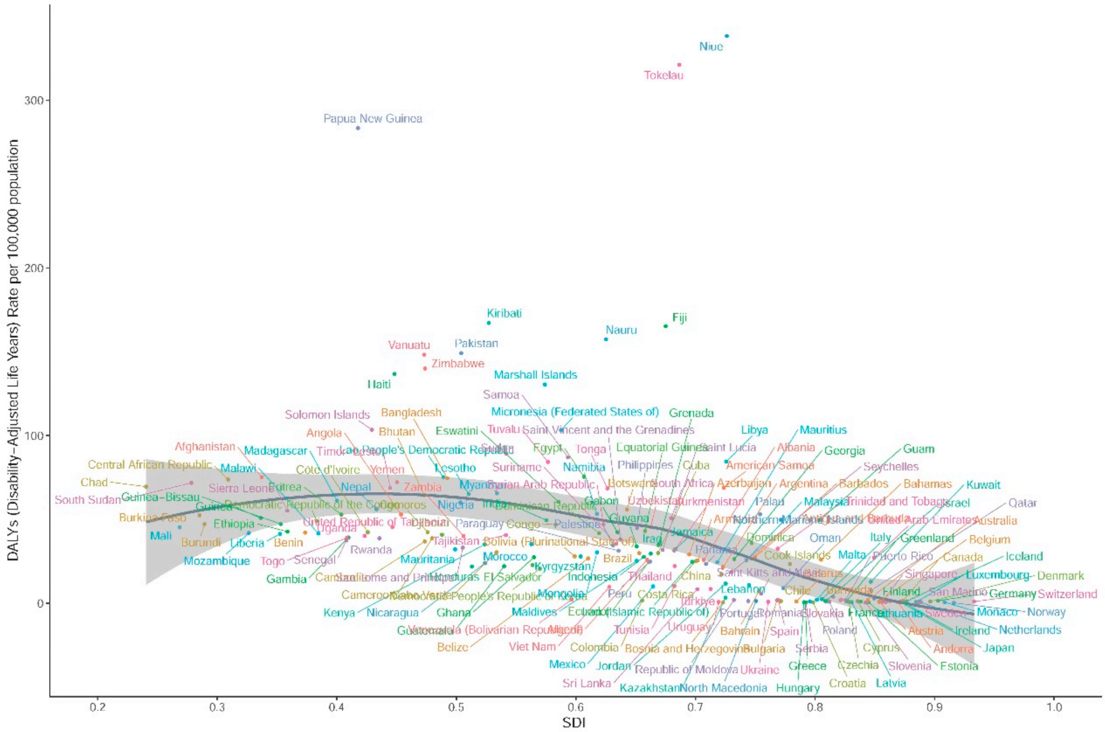

Figure S8 Country-level association between SDI and pediatric RHD DALYs rates in 2021.

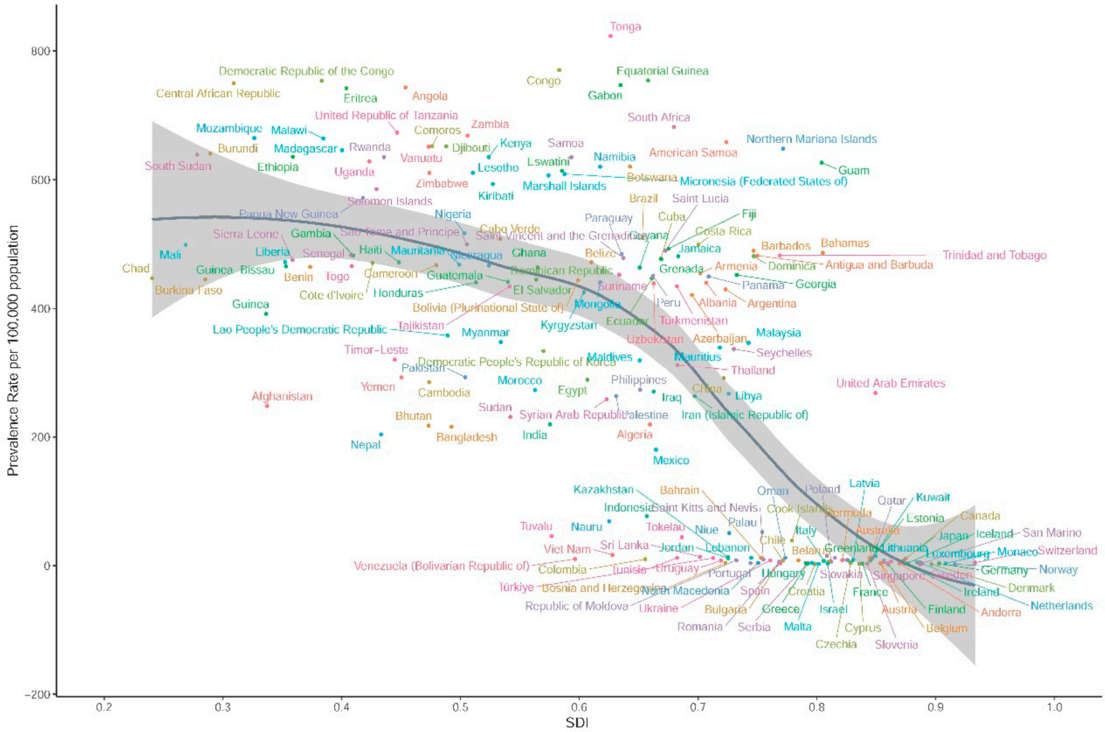

Figure S9 Country-level association between SDI and pediatric RHD prevalence rates in 2021

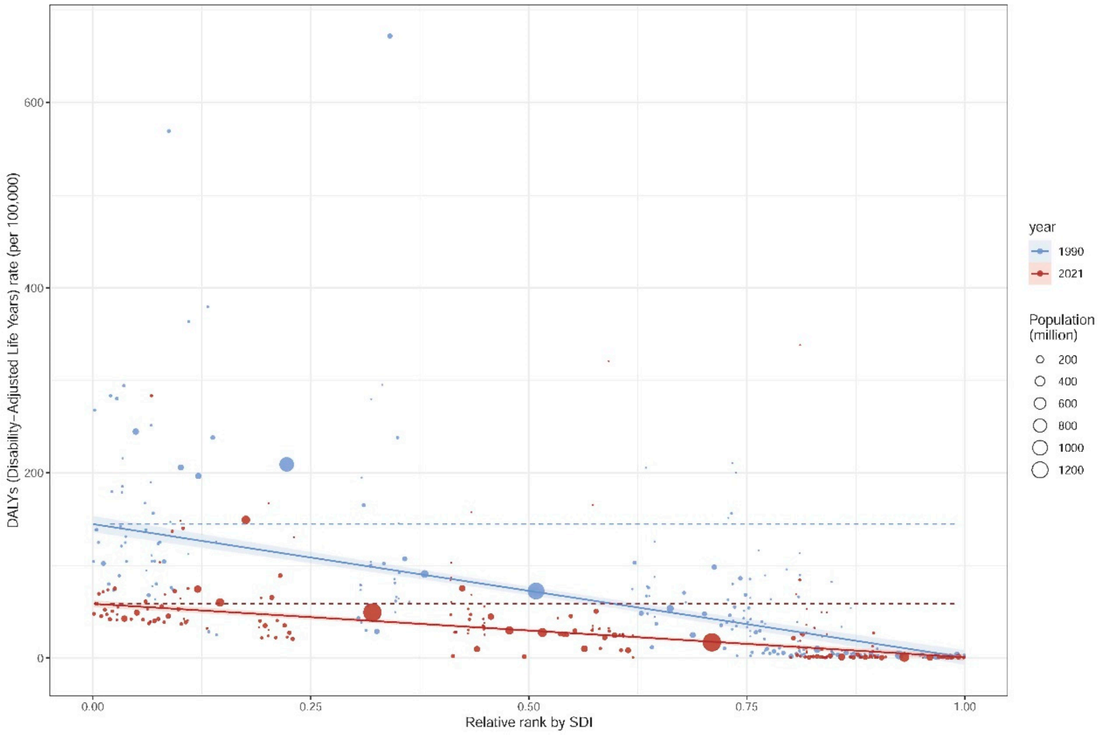

Figure S10 Slope indices of inequality for RHD-related DALYs in children aged 0–14 years, by SDI ranking, 1990 vs. 2021

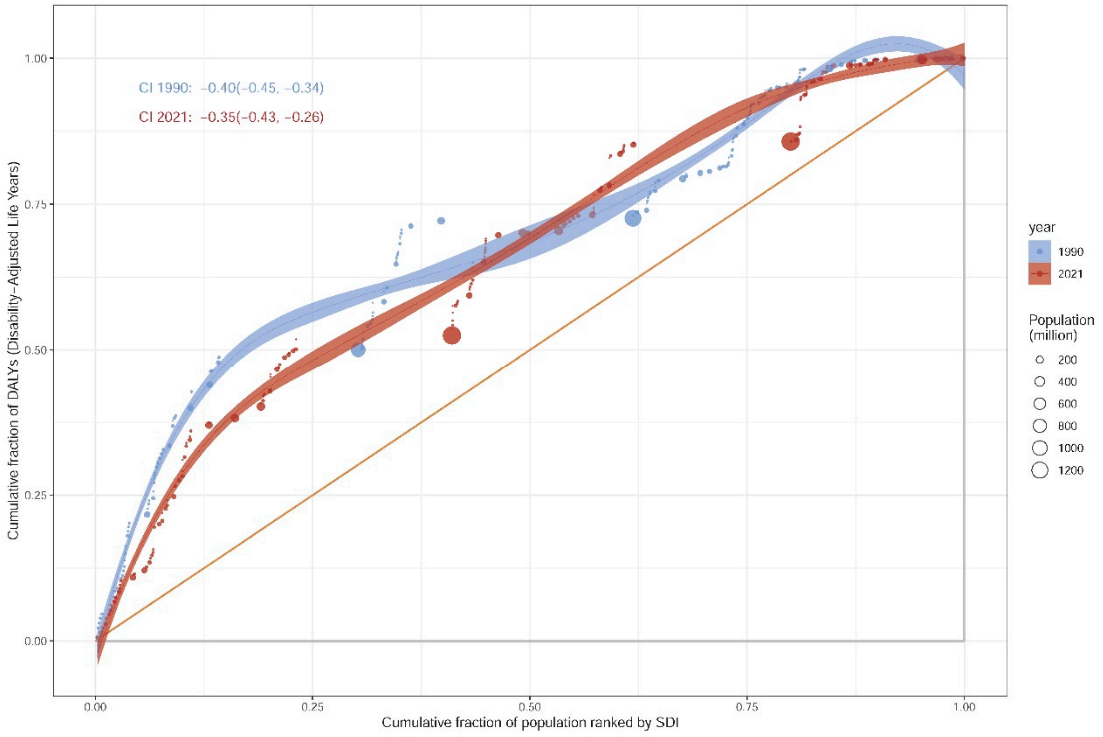

Figure S11 Concentration indices of inequality for RHD-related DALYs in children aged 0–14 years, by SDI ranking, 1990 vs. 2021

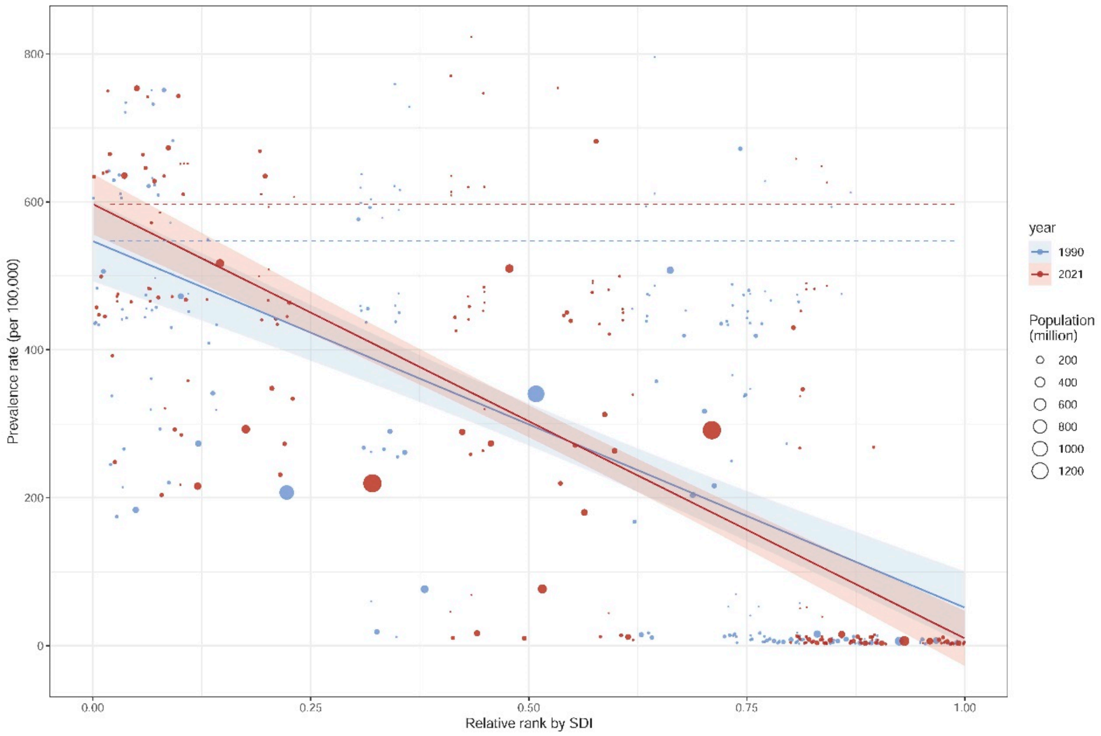

Figure S12 Slope indices of inequality for RHD-related prevalence, in children aged 0–14 years, by SDI ranking, 1990 vs. 2021

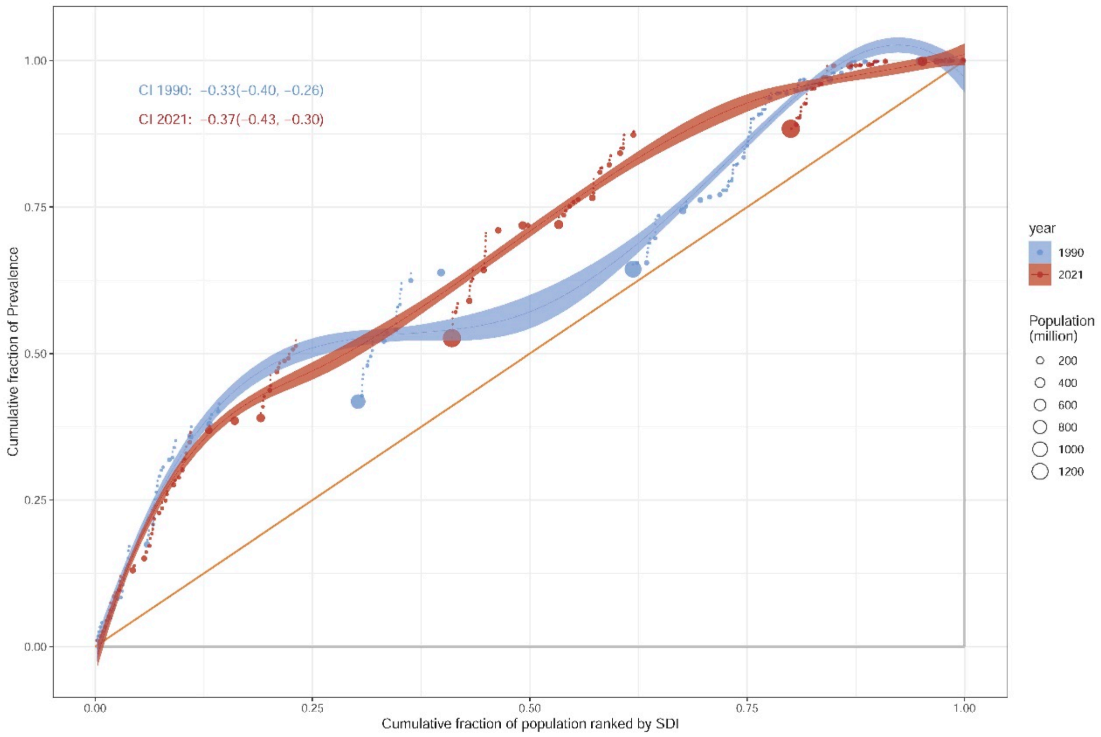

Figure S13 Concentration indices of inequality for RHD-related prevalence in children

aged 0–14 years, by SDI ranking, 1990 vs. 2021
