# Supplementary material for: Global Burden of Pediatric Rheumatic Heart Disease, 1990–2021: Analysis of the GBD 2021 Study
Source: Children (Basel). 2025 Jun 26;12(7):843. doi: 10.3390/children12070843 (PMC12293350; doi:10.3390/children12070843)
Supplement: Supplementary file 1 [file children-12-00843-s001.zip › Table S1.pdf]

Table S1 Age-standardized disability-adjusted life year rates of rheumatic heart disease among children aged 0–14 years, 1990–2021

Location name	1990 cases	1990 ASR of DALYs (per 100, 000, 95% UI)	2021 cases	2021 ASR of DALYs (per 100, 000, 95% UI)	AAPC (%, 95% CI)
Global	2044552.08(1627943.46,2634901.55)	117.22(90.07,154.09)	848879.08(668828.15,1076343.83)	41.56(31.65,54.84)	-3.30 (-3.41, -3.18)
High SDI	11838.32(10015.09,14192.41)	6.37(5.25,7.90)	2995.35(2470.35,3636.83)	1.69(1.39,2.08)	-4.08 (-4.29, -3.87)
High-middle SDI	97943.84(82348.75,116436.74)	35.77(29.52,43.98)	23553.50(17063.70,32582.44)	9.77(6.88,13.74)	-4.12 (-4.32, -3.92)
Middle SDI	498425.12(422687.42,583213.25)	86.27(71.29,104.48)	166794.40(127078.62,219792.74)	28.09(20.93,38.52)	-3.60 (-3.73, -3.47)
Low-middle SDI	967576.70(754430.68,1265143.95)	203.43(152.26,273.55)	366383.09(296626.21,454118.11)	62.24(48.86,79.33)	-3.75 (-3.89, -3.60)
Low SDI	467179.36(329176.17,665361.72)	196.97(135.51,279.97)	288059.67(212336.08,379369.98)	62.84(44.01,88.03)	-3.63 (-3.82, -3.43)
Andean Latin America	6074.40(4413.19,8324.68)	41.04(28.65,57.78)	4689.79(2858.65,7298.27)	25.48(15.07,40.60)	-1.49 (-1.61, -1.37)
Australasia	282.61(249.57,319.30)	6.07(5.21,7.09)	126.05(103.93,155.61)	2.12(1.70,2.66)	-3.31 (-3.84, -2.78)
Caribbean	15964.23(12096.39,20840.02)	139.36(95.32,193.01)	8576.73(6238.56,11314.66)	73.69(48.84,106.01)	-1.97 (-2.15, -1.79)
Central Asia	14178.20(11996.89,16808.93)	57.84(47.48,71.10)	8523.81(6290.02,11545.22)	31.35(22.50,43.40)	-1.96 (-2.25, -1.66)
Central Europe	2473.49(2278.81,2697.72)	8.26(7.46,9.21)	303.46(234.22,399.34)	1.64(1.24,2.17)	-4.93 (-5.21, -4.65)
Central Latin America	14081.34(11507.65,17978.44)	21.94(17.56,28.64)	7181.94(4386.13,11284.30)	10.69(6.21,17.12)	-2.27 (-2.40, -2.13)
Central Sub-Saharan Africa	29282.95(18834.14,41481.86)	113.89(64.30,175.50)	29371.78(18800.40,42727.59)	50.86(30.70,77.64)	-2.57 (-2.66, -2.49)
East Asia	234099.22(192595.59,287566.97)	70.72(55.43,88.87)	47649.63(29650.72,71932.76)	16.96(10.32,26.45)	-4.48 (-4.71, -4.25)
Eastern Europe	4113.46(3904.01,4386.44)	7.91(7.45,8.50)	481.54(373.43,621.86)	1.28(0.98,1.65)	-5.71 (-6.45, -4.96)
Eastern Sub-Saharan Africa	80036.00(56478.56,110741.51)	87.80(57.45,128.03)	78213.92(48995.16,113455.84)	44.18(26.37,68.11)	-2.19 (-2.30, -2.08)
High-income Asia Pacific	880.74(767.39,1011.75)	2.44(2.05,2.91)	174.16(140.80,217.09)	0.75(0.60,0.94)	-3.74 (-4.01, -3.48)
High-income North America	2173.27(2043.58,2343.37)	3.52(3.26,3.83)	830.59(712.56,977.57)	1.25(1.06,1.51)	-2.98 (-3.41, -2.56)
North Africa and Middle East	339807.94(236129.72,458824.73)	239.32(158.68,333.43)	86370.70(66079.55,115831.62)	47.11(34.47,65.82)	-5.09 (-5.20, -4.97)
Oceania	8323.07(5072.01,11985.20)	313.00(184.79,472.56)	12643.96(8887.10,17878.93)	252.61(166.05,371.38)	-0.65 (-0.98, -0.32)
South Asia	930328.09(683903.34,1290449.37)	213.31(149.54,298.05)	357205.23(284334.21,450876.78)	68.82(52.16,89.73)	-3.60 (-3.85, -3.35)
Southeast Asia	157039.79(122891.53,190549.12)	91.37(63.82,114.90)	57250.13(43009.79,70594.02)	31.92(23.62,39.99)	-3.34 (-3.52, -3.15)
Southern Latin America	3141.21(2160.71,4595.43)	20.85(14.11,30.87)	2433.25(1424.08,3749.01)	15.57(8.76,25.14)	-0.96 (-1.06, -0.86)
Southern Sub-Saharan Africa	18366.81(14128.54,23229.61)	89.42(62.28,119.62)	18303.43(13910.98,23693.99)	74.33(53.23,99.32)	-0.61 (-0.97, -0.26)
Tropical Latin America	29833.46(23473.25,38660.14)	53.52(41.78,70.13)	15320.91(9436.45,23352.90)	29.99(18.33,46.62)	-1.77 (-1.96, -1.58)
Western Europe	2677.70(2526.48,2869.68)	3.74(3.49,4.03)	865.91(753.73,1010.21)	1.24(1.06,1.46)	-3.39 (-3.77, -3.01)
Western Sub-Saharan Africa	151394.10(100173.56,218799.23)	160.86(100.55,237.84)	112362.17(74614.46,151763.24)	52.58(31.26,77.80)	-3.57 (-3.68, -3.45)

Abbreviations: UI – uncertainty interval; AAPC – average annual percentage change; CI – confidence interval; SDI – Socio-demographic Index.
