# Supplementary material for: Global Burden of Pediatric Rheumatic Heart Disease, 1990–2021: Analysis of the GBD 2021 Study
Source: Children (Basel). 2025 Jun 26;12(7):843. doi: 10.3390/children12070843 (PMC12293350; doi:10.3390/children12070843)
Supplement: Supplementary file 1 [file children-12-00843-s001.zip › Table S2.pdf]

Table S2. Age-standardized prevalence rate (ASPR) of rheumatic heart disease among children aged 0–14 years by global, regional, and SDI-level, 1990–2021

Location name	1990 cases	1990 ASPR (per 100, 000, 95% UI)	2021 cases	2021ASPR (per 100, 000, 95% UI)	AAPC (%, 95% CI)
Global	4366755.26(3001305.32,6068514.05)	253.82(167.38,366.88)	6226651.48(4235279.87,8726561.69)	298.55(194.94,435.24)	0.53 (0.45, 0.62)
High SDI	21345.12(16847.97,26094.13)	11.25(8.67,14.09)	21339.39(16765.94,26358.52)	11.84(9.17,14.82)	0.21 (0.07, 0.34)
High-middle SDI	383089.08(268515.77,519825.84)	136.97(93.98,193.39)	294712.43(206808.35,403438.54)	120.21(81.96,171.06)	-0.41 (-0.54, -0.29)
Middle SDI	1766680.83(1205378.62,2456357.01)	304.17(200.75,438.41)	1754272.62(1195652.25,2450394.94)	292.36(191.89,424.33)	-0.13 (-0.21, -0.06)
Low-middle SDI	1273069.94(859650.75,1772461.20)	279.54(181.43,406.50)	1937966.11(1306331.39,2728620.36)	322.33(207.96,470.50)	0.45 (0.33, 0.57)
Low SDI	919096.68(629253.81,1285901.30)	437.23(284.35,635.09)	2213480.50(1493243.98,3098127.37)	488.29(317.07,718.84)	0.36 (0.32, 0.40)
Andean Latin America	64404.59(43281.85,89933.01)	436.48(287.41,633.80)	82553.58(56095.68,114785.21)	448.16(292.95,650.25)	0.07 (0.05, 0.09)
Australasia	582.59(403.86,802.40)	12.50(8.37,17.90)	722.91(508.57,991.77)	12.20(8.37,17.05)	-0.06 (-0.10, -0.02)
Caribbean	46820.31(31895.50,65080.26)	414.60(272.09,597.86)	52796.22(36139.95,74575.99)	446.83(290.79,644.30)	0.24 (0.22, 0.26)
Central Asia	81605.39(56319.87,112441.23)	343.59(227.51,496.81)	94973.23(65622.71,132968.25)	352.00(232.82,508.54)	0.09 (0.07, 0.11)
Central Europe	6718.75(4978.81,8690.24)	21.45(15.52,28.23)	3041.01(2311.10,3864.97)	16.27(11.97,21.18)	-0.89 (-0.95, -0.83)
Central Latin America	122053.02(85492.04,169300.81)	190.68(127.92,271.49)	130338.51(90135.60,181414.89)	193.83(129.59,278.77)	0.05 (0.04, 0.07)
Central Sub-Saharan Africa	169805.32(114446.05,239804.23)	746.70(487.50,1085.15)	431662.13(288495.11,614888.48)	751.30(485.77,1094.51)	0.01 (-0.03, 0.04)
East Asia	1112794.69(760541.21,1534774.07)	334.75(222.55,477.83)	817734.08(566099.49,1125562.06)	289.06(192.52,416.93)	-0.40 (-0.52, -0.28)
Eastern Europe	7026.10(5480.84,8718.05)	13.46(10.29,17.26)	5072.72(3973.13,6252.00)	13.40(10.17,17.00)	-0.00 (-0.06, 0.05)
Eastern Sub-Saharan Africa	491987.13(335490.37,689706.08)	589.79(385.46,856.52)	1142738.69(764500.08,1613615.26)	647.34(419.88,947.38)	0.34 (0.25, 0.43)
High-income Asia Pacific	2501.07(1855.06,3333.17)	6.76(4.85,9.19)	1385.84(1062.68,1763.98)	5.95(4.52,7.65)	-0.40 (-0.45, -0.34)
High-income North America	3916.19(2951.33,5124.84)	6.35(4.62,8.57)	4437.52(3492.70,5591.96)	6.56(5.04,8.39)	0.19 (-0.04, 0.42)
North Africa and Middle East	264298.76(183141.56,360464.97)	193.31(129.06,277.89)	397375.92(269914.28,550779.91)	212.31(138.35,308.53)	0.29 (0.21, 0.38)
Oceania	13868.49(9968.25,18538.75)	536.17(373.22,754.80)	27838.57(19831.89,37376.42)	572.61(396.45,806.34)	0.22 (0.18, 0.27)
South Asia	886526.25(585993.46,1264529.72)	211.43(133.85,310.42)	1251437.39(807940.45,1798140.21)	230.44(143.91,339.71)	0.32 (-0.01, 0.64)
Southeast Asia	264639.97(184458.41,358005.49)	153.00(103.81,217.77)	299134.38(206995.97,411330.45)	166.33(111.60,237.79)	0.30 (0.26, 0.35)
Southern Latin America	43753.12(30420.49,59771.18)	289.92(194.33,412.19)	47633.83(32941.04,65397.65)	304.36(204.17,434.57)	0.15 (0.13, 0.17)
Southern Sub-Saharan Africa	131505.57(88492.35,182491.03)	646.99(425.40,940.21)	162641.12(109252.37,227916.72)	656.87(433.29,950.87)	0.04 (0.01, 0.06)
Tropical Latin America	284256.66(191652.23,402514.96)	506.67(333.41,736.68)	260053.71(175641.58,367288.94)	508.85(335.52,740.02)	0.01 (0.01, 0.02)
Western Europe	3448.03(2426.49,4681.88)	4.73(3.26,6.65)	2722.79(1923.07,3680.55)	3.88(2.70,5.38)	-0.53 (-0.73, -0.34)
Western Sub-Saharan Africa	364243.26(248154.99,504381.20)	457.39(299.00,666.99)	1010357.32(681023.04,1406541.95)	489.10(319.45,713.43)	0.21 (0.20, 0.22)

Abbreviations: UI – uncertainty interval; AAPC – average annual percentage change; CI – confidence interval; SDI – Socio-demographic Index.
